# BcsZ inhibits biofilm phenotypes and promotes virulence by blocking cellulose production in *Salmonella enterica* serovar Typhimurium

**DOI:** 10.1186/s12934-016-0576-6

**Published:** 2016-10-19

**Authors:** Irfan Ahmad, Syed Fazle Rouf, Lei Sun, Annika Cimdins, Sulman Shafeeq, Soazig Le Guyon, Marco Schottkowski, Mikael Rhen, Ute Römling

**Affiliations:** 1 Department of Microbiology, Tumor and Cell Biology, Karolinska Institutet, Stockholm, Sweden; 2 Department of Molecular Biology, Umeå University, Umeå, Sweden; 3 Département de Biologie, Faculté des Sciences, Université de Sherbrooke, Quebec, Canada

**Keywords:** Cellulose, Cellulase, BcsZ, Biofilm, CsgD, *Salmonella*

## Abstract

**Background:**

Cellulose, a 1,4 beta-glucan polysaccharide, is produced by a variety of organisms including bacteria. Although the production of cellulose has a high biological, ecological and economical impact, regulatory mechanisms of cellulose biosynthesis are mostly unknown. Family eight cellulases are regularly associated with cellulose biosynthesis operons in bacteria; however, their function is poorly characterized. In this study, we analysed the role of the cellulase BcsZ encoded by the *bcsABZC c*ellulose biosynthesis operon of *Salmonella enterica* serovar Typhimurium (*S.* Typhimurium) in biofilm related behavior. We also investigated the involvement of BcsZ in pathogenesis of *S.* Typhimurium including a murine typhoid fever infection model.

**Result:**

In *S.* Typhimurium, cellulase BcsZ with a putative periplasmic location negatively regulates cellulose biosynthesis. Moreover, as assessed with a non-polar mutant, BcsZ affects cellulose-associated phenotypes such as the rdar biofilm morphotype, cell clumping, biofilm formation, pellicle formation and flagella-dependent motility. Strikingly, although upregulation of cellulose biosynthesis was not observed on agar plate medium at 37 °C, BcsZ is required for efficient pathogen-host interaction. Key virulence phenotypes of *S.* Typhimurium such as invasion of epithelial cells and proliferation in macrophages were positively regulated by BcsZ. Further on, a *bcsZ* mutant was outcompeted by the wild type in organ colonization in the murine typhoid fever infection model. Selected phenotypes were relieved upon deletion of the cellulose synthase BcsA and/or the central biofilm activator CsgD.

**Conclusion:**

Although the protein scaffold has an additional physiological role, our findings indicate that the catalytic activity of BcsZ effectively downregulates CsgD activated cellulose biosynthesis. Repression of cellulose production by BcsZ subsequently enables *Salmonella* to efficiently colonize the host.

**Electronic supplementary material:**

The online version of this article (doi:10.1186/s12934-016-0576-6) contains supplementary material, which is available to authorized users.

## Background

Cellulose production is not only of high economic impact for the wood processing and food industry, but also of biological importance in the fields of medicine, agriculture and ecology [1]. The exopolysaccharide cellulose is produced by trees, other plants, amoeba, fungi and animals, but also by numerous bacteria from diverse branches of the phylogenetic tree such as the Thermotoga, cyanobacteria, rhizobia and proteobacteria [1–8]. Among the gammaproteobacteria, the enterobacterial pathogens *Salmonella enterica* serovar Typhimurium (*S.* Typhimurium), *Escherichia coli* (*E. coli*) and *Klebsiella pneumoniae* produce cellulose [1, 9, 10].

The biological roles of cellulose biosynthesis are manifold. In bacteria, cellulose is a major structural component, which provides cell-surface and cell–cell interaction in different biofilm models [9, 11] and protects from chlorine treatment [12]. In *S.* Typhimurium, cellulose is a major component of the extracellular matrix of the red, dry and rough (rdar) morphotype, a colony biofilm behavior. The major biofilm activator CsgD positively regulates predominant rdar extracellular matrix components amyloid curli fimbriae and cellulose [13]. Further on, cellulose is an extracellular matrix component of pellicle, flow cell and other types of biofilms [11].

In addition, deregulated cellulose production alters bacterial-eukaryotic host interactions. For example, cellulose biosynthesis affects the interaction between commensal and pathogenic *E. coli* and *S.* Typhimurium and intestinal epithelial cells and is produced inside macrophages to reduce virulence [14–17], suggesting that tight regulation of this matrix component contributes to an effective infection process [14, 18]. In plant-associated bacteria, cellulose mediates the interaction between bacteria and plant roots facilitating tight adherence [19, 20].

**Fig. 1:**
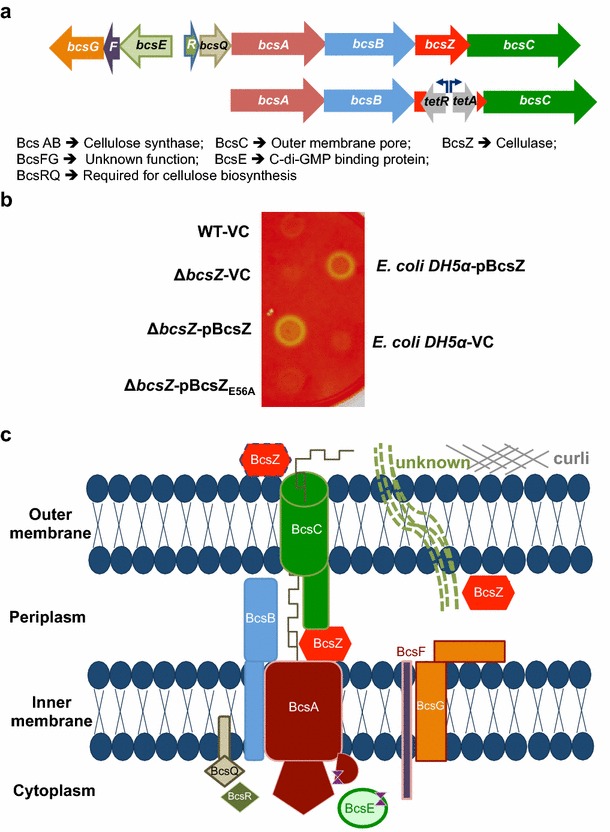
The cellulose biosynthesis operon, gene organization, proteins and functions. **a** *Upper line* Organization of the cellulose biosynthesis operon *bcsEFG*-*bcsRQABZC* in *S.* Typhimurium. *bcsA* and *bcsB* encode the cellulose synthase and *bcsZ* encodes a cellulase. *bcsEFG* and *bcsR* are characteristic for class II cellulose operons, while *bcsQ* is also found in class I operons [25]. *Lower line* Construction scheme of the non-polar *bcsZ* mutant using the *tetRA* gene cassette. **b** Detection of cellulase activity upon deletion and overexpression of BcsZ in *S.* Typhimurium UMR1 wildtype (WT). Bacterial cells were grown on carboxymethyl (CMC)-containing LB without salt agar plates.* Yellow spots* indicate cellulase activity through CMC degradation. Residual cellulase activity is seen in the wild type UMR1. BcsZ overexpression shows pronounced cellulase activity, abolished in the catalytic mutant BcsZ_E56A_. Positive control *E. coli* DH5α pBcsZ and negative control *E. coli* DH5α VC. VC = pBAD30; pBcsZ = BcsZ cloned in pBAD30; pBcsZ_E56A_ = BcsZ_E56A_ cloned in pBAD30.** c** The cellulose secretion apparatus of *S.* Typhimurium modified after [25]. BcsA and BcsB form the active cellulose synthase complex. BcsC is supposed to be a pore in the outer membrane. BcsZ is a cellulase potentially located in the periplasm, but is found secreted in other cellulose producing/non-producing bacteria. Curli might aid the production of another unknown periplasmic/extracellular component requiring BcsZ. BcsE is a c-di-GMP binding protein required for optimal cellulose biosynthesis. The function of BcsF and BcsG is unknown. BcsQ and BcsR are also required for cellulose biosynthesis

In Enterobacteria, cellulose biosynthesis is directed by the *bcsABZC* operon (Fig. 1a). Thereby, *bcsA* encodes the catalytic subunit of the cellulose synthase with the cytoplasmic beta-glycosyltransferase 2 domain, which binds the substrate UDP-glucose [21]. BcsB is required for catalytic activity and consistently co-localizes with *bcsA* whereby in some strains a BcsAB fusion protein is formed [22, 23]. BcsC is suggested to form an outer membrane pore (Fig. 1c; [24]). Three types of confirmed cellulose biosynthesis operons exist containing distinct accessory genes in addition to the core *bcsAB* genes [25]. The cellulose biosynthesis operon of *S*. Typhimurium belongs to type IIa [25], characterized by the presence of divergently transcribed *bcsEFG* genes (Fig. 1a, c). Thereby, BcsE is required for optimal cellulose biosynthesis [26]. BcsZ encodes a cellulase of family 8 glycoside hydrolases (GH8) [27] with unknown biological function in cellulose biosynthesis in bacteria harboring the *bcsABZC* operon.

Little is known about the regulation of cellulose biosynthesis. It is the common view that the biosynthesis operon is transcribed constitutively [28]; enhanced transcription in the stationary phase of growth was observed in *S.* Typhimurium [9]. Beyond transcription, the cellulose macromolecule is not constitutively produced. A major regulatory pathway on the post-translational level is the secondary messenger cyclic di-GMP (c-di-GMP), whereby the molecule binds to the C-terminal PilZ domain of the cellulose synthase BcsA and the GIL receptor BcsE to activate and optimize cellulose biosynthesis, respectively [26, 29]. C-di-GMP dedicated to cellulose biosynthesis is produced by the di-guanylate cyclase AdrA. The activity of AdrA is mainly regulated on the transcriptional level by the orphan response regulator CsgD positively affecting *adrA* [30].

In this manuscript, we investigate the role of the cellulase BcsZ in cellulose biosynthesis. We show that BcsZ potentially locates in the periplasm and downregulates cellulose biosynthesis, which subsequently affects biofilm formation, motility and pathogen-host interactions. Indeed, BcsZ contributes to, for example, increased intracellular proliferation in macrophages as well as organ colonization in a systemic infection model of mice. Most, but not all phenotypes of a *bcsZ* deletion are relieved upon deletion of the cellulose synthase BcsA and/or the biofilm activator CsgD suggesting that BcsZ deregulates *csgD* mediated cellulose biosynthesis to affect a broad range of phenotypes.

## Methods

### Bacterial strains, plasmids and growth conditions

Bacterial strains and plasmids used in this study are listed in Additional file 1. For genetic manipulations, *E. coli* Top10 and DH5α and *S.* Typhimurium UMR1 (ATCC14028 Nal^r^; rdar_28_) were grown on Luria–Bertani (LB) agar plates or broth overnight at 37 °C. To induce the invasion phenotype, *S. *Typhimurium was grown overnight in LB  broth + 0.3 M NaCl in standing culture, diluted 1:100 in fresh medium and grown until OD_600_  =  0.6. Antibiotics were ampicillin (100 μg ml^−1^), kanamycin (30 μg ml^−1^) and chloramphenicol (20 μg ml^−1^). For induction of *bcsZ* cloned in pBAD30, 0.1 % L-arabinose was used, if not otherwise stated.

### Construction of mutants

*BcsZ* mutants were generated by homologous recombination [31] replacing the open reading frame (ORF), except for 40 nucleotides from the start and stop codon. For the non-polar *bcsZ* mutant, the *tetRA* cassette along with *bcsZ* homologous overhangs was PCR-amplified from *S.* Typhimurium TT946 and electroporated into *S. *Typhimurium UMR1 carrying pKD46 (primers in Additional file 2). Recovered colonies were purified at least twice on LB medium containing the corresponding antibiotics.

A 3xFLAG-tagged *bcsC* was constructed using the lambda-red recombination system [31]. The 3xFLAG-Km was amplified from pSUB11 [32] and the final construct was verified by sequencing.

Phage transduction of mutant alleles into a novel strain background was carried out with phage P22 HT105/1 *int*-*201*. Transductants were colony purified twice on LB agar plates containing 10 mM EGTA and appropriate antibiotics. All constructed mutants were verified by PCR with control primers located in the genes flanking the deleted ORF.

### Plasmid construction

*BcsZ* was amplified using primer pair BcsZclon_N and BcsZclon_C (Additional file 2) from template *S. *Typhimurium UMR1. The resulting PCR product was digested with restriction endonucleases *Sac*I and *Sph*I, and ligated with *Sac*I/*Sph*I-restricted vector pBAD30. The ligation product was transformed into *E. coli* DH5α. The BcsZ_E56A_ mutant was constructed by overlapping PCR using primers containing the mutation (Additional file 2). Gene integrity was verified by DNA sequencing.

### Phenotypic evaluation—Rdar morphotype assay

Five microliters of an overnight culture suspended in PBS (OD_600_ of 5) were spotted onto LB without salt agar plates supplemented with Congo red (40 μg ml^−1^) and Coomassie Brilliant Blue (20 μg ml^−1^) and incubated at 28 °C for up to 48 h. The development of the colony morphology and dye binding was analysed over time. The rdar morphotype indicates expression of the extracellular matrix components cellulose and amyloid curli fimbriae, while a pink, dry and rough (pdar) morphotype indicates cellulose production only. Control strains with expression of curli only, Δ*bcsA*, and without a distinct morphotype, Δ*csgD*, are brown, dry and rough (bdar) and smooth and white (saw), respectively.

### Calcofluor binding assay

For a qualitative agar plate assay, five microliters of an overnight culture suspended in PBS (OD_600_ of 5) were spotted onto LB without salt agar plates supplemented with Calcofluor (fluorescence brightener 28). Plates were incubated at 28 °C for up to 48 h and dye binding was documented at distinct time intervals.

The amount and distribution of cellulose was also assessed by fluorescent microscopy. LB without salt agar plates were incubated at 28 °C for 48 h and bacterial cells from the middle of the colony gently resuspended in 10 μg/ml Calcofluor dissolved in water. Cellulose production indicated by fluorescence intensity and cell aggregation was observed with an Olympus FV1000 confocal microscope.

A quantitative agar plate assay was performed as described [26]. Briefly, plate-grown cells were suspended to an optical density of OD_600_ = 0.1. Eight microliter was added into each well of a black 96-well microtiter plate with clear bottom (BD falcon) filled with 200 μl LB without salt agar containing 50 μg ml^−1^ Calcofluor, 0.1 % L-arabinose and 100 μg ml^−1^ ampicillin. After 24 h and 48 h incubation at 28 °C, the emission intensity at 460 nm was recorded (excitation at 355 nm) with a multilabel reader (VICTOR™ X3, Perkin Elmer).

### Biofilm formation in M9 medium

To assess cellulose production in M9 minimal medium, bacteria grown on LB plates overnight were suspended in PBS. The suspension was inoculated in M9 medium adjusted to OD_600_ = 0.1. The culture was incubated at 28°C for 24 h with 200 rpm shaking after which bacterial clumping is indicative for cellulose production.

### Pellicle formation

In *S.* Typhimurium, pellicle formation, air–liquid interface growth, in standing culture requires cellulose production. After overnight growth at 37 °C, 20 µl of the pre-culture was used to inoculate 180 µl of saltless LB broth in 96 well plates. The plate was incubated at 28 °C for 48 h. Pellicle strength was determined by subsequent addition of glass beads (Ø 0.75–1.00 mm, Retsch) using a tweezer until disruption.

### Swimming and swarming motility

Swimming motility was observed in 0.3 % LB agar plates incubated for 5 h at 28 °C and 4 h at 37 °C after inoculation with a single colony from an overnight LB plate culture at 37 °C.

Swarming motility was analysed on 0.5 % LB agar plates supplemented with 0.5 % glucose at 28 and 37 °C after inoculation with a single colony from an overnight LB plate culture at 37 °C. The radius from the inoculation point to the edge of the motility zone was measured after 4 h. All experiments were done at least twice in duplicates.

#### Cellulase assay

To demonstrate cellulase activity of BcsZ, 5 µl of a suspension of *S*. Typhimurium UMR1 and derivatives in PBS (OD_600_ of 5), grown on LB plates overnight, were spotted onto LB without salt agar plates supplemented with 5 % carboxy methyl cellulose. Colonies were removed from the plate after 48 h incubation at 28 °C. 0.1 % Congo red was spread on the plates, incubated for 30 min at room temperature and plates were washed 3 times with 0.9 % NaCl for 15 min. A yellow spot on the red plate background indicated cellulase activity.

### Flow cell biofilm experiment

Single colonies were inoculated in 3 mL of LB for overnight growth at 37 °C, 200 rpm. Cultures were adjusted to an OD_600_ of 0.04 in M9 minimal medium with 0.4 % glucose, 100 μg ml^−1^ ampicillin and 0.01 % L-Arabinose. The channels of BioFlux 48 well plate were primed with M9 medium with 0.4 % glucose. A bacterial suspension was seeded into the channels starting from the output side at 2 dyn/cm^2^ for 3 s and incubated at 28 °C for 1 h to allow attachment. The liquid in outlet well was removed, 900 µl fresh medium added to inlet well with flow 0.58 dyn/cm^2^ for 19 h. To terminate biofilm formation, 500 µl 70 % isopropyl alcohol was added to inlet well with flow of 0.58 dyn/cm^2^ for 1 h. After fixation, cells were stained with 25 µl propidium iodine added to inlet well at 0.55 dyn/cm^2^ for 5 min. After 15 min incubation in the dark, the fluorescence images were acquired with Zeiss LSM510META Confocal Microscope with a 10× objective.

### Fitness experiment in LB medium

Overnight bacterial plate cultures of wild type and mutant were suspended and mixed in a 1:1 ratio in PBS. Approximately 10^3^ cells were added to 50 ml LB broth to incubate with shaking (220 rpm) at 37 °C. After 6 and 16 h of growth, cell numbers were estimated by differentially plating 10-fold dilutions onto LB agar plates ± appropriate antibiotics for estimation of viable counts (cfu). The competitive index (CI) of wild type towards mutant was calculated as described [33].

### Protein localisation assays

To assess whether BcsZ is secreted, cells were grown in LB medium (35 ml in a 50 ml flask) up to OD_600_ 1.5. Aliquots of 1.5 ml supernatant and cell-associated protein from 0.3 ml suspension were analysed for BcsZ expression by Western blot analysis after trichloroacetic acid precipitation.

To assess surface association of BcsZ, proteinase K digestion of whole bacterial cells was performed. In brief, 10 mg of bacteria were harvested after 48 h of growth on LB without salt plates at 28 °C. Cells were resuspended in 1 ml of Tris–HCl buffer pH 7.5, 5 mM CaCl_2_ and 40 µg/ml chloramphenicol. Aliquots of the bacterial suspension were digested with different concentrations of proteinase K for 2 h at 37 °C. The suspension was adjusted to 10 % trichloroacetic acid and incubated on ice for 30 min. The reaction mixture was centrifuged and the pellet washed three times with 70 % ethanol. The dried pellet was reconstituted in 80 µl SDS sample buffer, boiled for 5 min and loaded on the gel. After protein separation, western blot analysis was performed to detect BcsZ, OmpR (cytoplasmic control protein) and DsbA (periplasmic control protein).

### Creation of an antibody against BcsZ

The conserved amino acid sequences specific for BcsZ, KKDYISQQGRVIDPGDARK and DWVRYESKQGWQLKAEK, were synthesized inhouse (Helmholtz Center for Infection Research, Braunschweig, Germany). The two peptides were used for production of a polyclonal antiserum in mice (Neosystem Group SNPE, France). The serum was loaded onto CnBr-activated Sepharose with peptides containing a N-terminal cysteine residue coupled. The column was washed with 0.1 M acetate buffer and fractions were eluted with 0.2 M acetate buffer and immediately neutralized with 1.5 M Tris–HCl, pH 8.8. In a second step, fractions were eluted with 100 mM Triethylamine buffer, pH 11.5 and neutralized immediately with 1.5 M Tris–HCl, pH 4.5. The fractions containing proteins were combined, buffer exchanged to PBS using Centricon-columns YM-30 and the antibody concentrated to 2 mg/ml.

### SDS-PAGE and Western blot analysis

Cell extracts were separated on an 4/8 % SDS-PAGE gel and electro-transferred onto a PVDF membrane (Millipore Corp.) at 120 mA for 4 h. Membranes were blocked using 5 % BSA and 5 % non-fat dry milk in TBST [20 mM Tris–HCl (pH 7.5), 150 mM NaCl and 0.05 % Tween-20] overnight. Anti-BcsZ peptide antibody was used at 1:3000 dilution. Detection of CsgD was carried out using polyclonal anti-CsgD peptide antibody (1:5000) as the primary antibody [30]. Anti-OmpR and anti-DsbA antibodies were used as previously described. Goat anti-rabbit immunoglobulin G (Jackson ImmunoResearch Laboratories) conjugated with horseradish peroxidase at a 1:5000 or 1:2000 dilution, respectively, was the secondary antibody. FLAG primary antibody (Sigma) was used at 1:2000 dilution with peroxidase-conjugated AffiniPure Goat Anti-Mouse IgG (Jackson ImmunoResearch) secondary antibody at 1:3000 dilution. After washing, binding of antibody was detected using the ECL light detection reagent (Roche). Visualization of bands was performed using FUJI LAS1000-plus chemiluminescence imaging system (Fuji, Stamford, CT, USA).

### Analysis of curli fimbriae expression

The major subunit of curli fimbriae, the CsgA protein, was enriched, subsequently treated with formic acid and detected on a protein gel [8]. Briefly, 3 mg of an overnight culture grown on LB without salt plate at 28 °C was resuspended in PBS and centrifuged. The pellet was re-suspended in TE buffer (10 mM Tris, 1 mM EDTA and 0.2 % SDS; pH = 7.5), boiled for 45 min at 95 °C and centrifuged at 14,000 rpm. The pellet was washed with H_2_O two times and dried in a Speed Vac for 1 h. The semi-purified curli were taken up in 100 % formic acid, incubated on ice for 15–20 min and formic acid was evaporated. The denatured pellet was dissolved in 200 μl SDS sample buffer, boiled for 15 min at 95 °C and loaded on a 15 % SDS-PAGE gel. CsgA was visualized by Coomassie staining of the gel.

### Human epithelial cell invasion assay

The human epithelial cell line HT-29 (ATCC HTB 38, colon, colorectal adenocarcinoma) was grown to confluence in 24-well plates in RPMI-1640 medium (Life Technologies) supplemented with 25 mM HEPES, 2 mM l-glutamine and 10 % fetal calf serum (Sigma/Aldrich) at 37 °C in 5 % CO_2_. Bacteria were diluted and seeded on confluent HT-29 cells grown in 24-well plates at a multiplicity of infection of 1.7, which corresponds to 10^7^ cfu ml^−1^. One-hour post infection, medium containing gentamicin at 100 μg ml^−1^ was added for 1 h to kill extracellular bacteria. Cells were gently washed twice with PBS and disrupted with 1 % Triton X-100 (Sigma Chemical) in PBS. The number of intracellular bacteria was determined by estimation of colony-forming units (cfu) on agar plates. An Δ*ompR* mutant was used as a negative control [14]. The invasion rate is defined as (cfu recovered inside cells after 1 h/cfu at time of inoculation). The relative invasion rate in % is defined as (invasion rate of mutant/invasion rate of wild type) * 100. Presented results are based on at least three biological replicates consisting of four technical replicates each.

### Macrophage infection assay

The murine RAW264.7 macrophage cell-line was cultured in RPMI medium (Gibco, UK) supplemented with 10 % fetal bovine serum (Gibco), 10 mM l-glutamine (Sigma), 10 mM HEPES (Sigma). Overnight bacterial plate cultures were opsonized in 10 % pre-immune BALB/c mouse serum for 30 min at 37 °C prior to infection at a MOI of 10. The macrophages were activated overnight with 10 ng/ml IFN-γ prior to infection. The uptake and intracellular proliferation rate of bacteria was assessed after 2 and 16 h, respectively, counting gentamycin protected bacteria by viable counts (cfu) after hypertonic lysis of macrophages [34] For the competition experiment, strains were mixed at a ratio of 1:1 prior to infection. The competitive index (CI) was calculated as described [33].

### Mouse experiments

Competition experiments between wild type and mutants were performed in 6–8 week old female BALB/c J mice (Taconic, Denmark). Overnight bacterial plate cultures were mixed at a 1:1 ratio in PBS and approximately 10^8^ cells/100 μl were administered orally. Livers and spleens of 5 mice/group were collected on day 1 and 3 post infection, homogenized and plated onto LB agar plates ± appropriate antibiotics for estimation of viable counts (cfu). Competitive index (CI) of wild type towards mutant was calculated as described [33]. Experiments were performed at the Department of Microbiology, Tumor and Cell Biology (MTC) animal facility, Karolinska Institutet, Stockholm, Sweden in accordance with national and institutional guidelines (ethical permit N133/13).

### Phylogenetic analysis

BcsZ was compared to all experimentally verified cellulases of family 8 glycosidases from the CAZy database (http://www.cazy.org/Citing-CAZy.html; [35]) and cellulases representative for the different classes of cellulose biosynthesis operons [25]. Protein sequences were aligned with Clustal X 2.1 using standard parameters, alignments manually curated and the tree was drawn with TreeView version 1.6.6.

### Statistical analysis

Prism 5 (GraphPad Software) was used to calculate statistics. Statistical analysis was performed using a paired Student’s *t* test or using the Kruskal–Wallis assessment with subsequent Dunn’s test.

## Results

### BcsZ has cellulase activity

The cellulase BcsZ encoded by the third gene of the cellulose biosynthesis operon *bcsABZC* of *S.* Typhimurium overlaps with the downstream *bcsC* by 19 bps (Fig. 1a). We created a non-polar mutant of *bcsZ* in the wild type strain *S.* Typhimurium UMR1 by replacing the open reading frame of *bcsZ* by the *tetRA* cassette with the *tetA* promoter outward to ensure expression of the downstream *bcsC* gene (Fig. 1a). An agar plate assay of carboxymethyl-cellulose (CMC) degradation has been used to indicate cellulase activity in *E. coli* and *Salmonella* by overexpression of BcsZ [27, 36]. After staining the plate with Congo red, a light halo around the colony is indicative for cellulase activity. Using this CMC degradation assay, a slight difference in cellulase activity between *S.* Typhimurium UMR1 and its *bcsZ* mutant was observed indicating residual activity (Fig. 1b). We next cloned *bcsZ* in plasmid pBAD30 under the control of an arabinose inducible promoter. Overexpression of BcsZ in the UMR1 *bcsZ* mutant background showed significant CMC-degradation capability. The glutamate E56 is required for the catalytic activity of the cellulase BcsZ [27]. In contrast, CMC-degradation was not observed upon overexpression of BcsZ_E56A_ in the UMR1 *bcsZ* mutant background. In conclusion, degradation of CMC by BcsZ, but not BcsZ_E56A_ demonstrates the cellulase activity.

### BcsZ decreases rdar morphotype development and increases Calcofluor binding

**Fig. 2:**
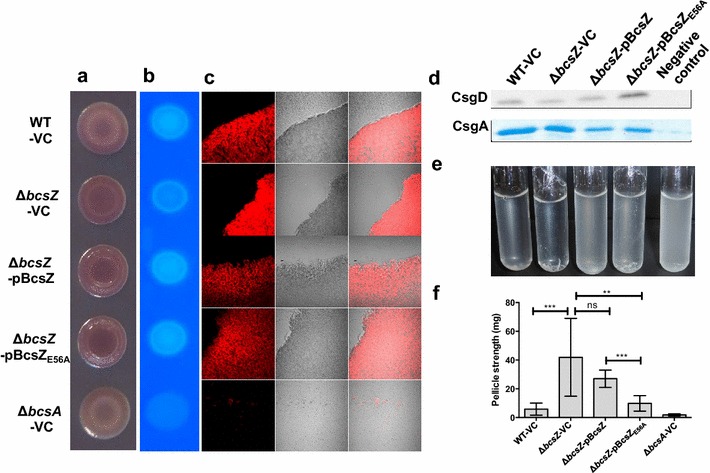
Biofilm phenotypes of the *bcsZ* deletion mutant of *S.* Typhimurium UMR1. **a** Rdar morphotype formation and **b** Calcofluor (CF) binding of *S.* Typhimurium UMR1 wildtype (WT) is enhanced upon deletion of *bcsZ*. Interestingly, overexpression of BcsZ did not complement the phenotype. **c** Calcofluor staining of colonies grown on agar plates indicates higher cellulose production in the *bcsZ* mutant. Overexpression of BcsZ complemented the phenotype, while overexpression of the catalytic mutant BcsZ_E56A_ showed a more patchy distribution of Calcofluor staining. **d** Expression of the biofilm regulator CsgD and the subunit of curli fimbriae CsgA is not altered upon deletion of *bcsZ*. **e** Cell clumping and biofilm formation of *S.* Typhimurium WT is enhanced upon deletion of *bcsZ* upon growth in M9 minimal medium for 16 h. Overexpression of BcsZ, but not of the catalytic mutant BcsZ_E56A_ complemented the phenotype. **a**, **c**, **d**, **e**: *S.* Typhimurium WT and derivatives were grown on LB without salt agar plates for 48 h at 28 °C. B: *S.* Typhimurium WT and derivatives were grown on LB without salt agar plates for 16 h at 28 °C. **f** Pellicle strength of *S.* Typhimurium in standing culture is enhanced upon deletion of *bcsZ*. *S*. Typhimurium WT and derivatives were grown in LB without salt standing culture for 48 h at 28 °C. Shown is a representative experiment with n = 24 for WT-VC and Δ*bcsZ*-VC and n = 6 technical replicates for the other derivatives. *Error bar* indicates SD. ***p < 0.0005, **p<0.001, *p < 0.05; *ns* not significant using Student’s paired t-test. Sample order remains for **a**–**c** (as shown on the *left panel*) and **d**–**e** (as shown on the *top panel*). *VC* pBAD30; *pBcsZ* BcsZ cloned in pBAD30; *pBcsZ*_*E56A*_ BcsZ catalytic mutant cloned in pBAD30. Δ*csgD*-VC and Δ*bcsA*-VC serves as negative controls for **d** and **e** respectively

To monitor the effect of BcsZ on cellulose production, the *bcsZ* mutant was compared with wild type UMR1 in rdar morphotype development (Fig. 2a). In UMR1, the rdar extracellular matrix components cellulose and curli fimbriae are tightly regulated, with optimal expression on LB without salt medium agar plates at 28 °C under microaerophilic conditions [37]. Most visible after 48 h of growth, the rdar morphotype of the *bcsZ* mutant was more developed compared to the wild type UMR1 suggesting higher cellulose production. Contrary to expection, though, the *bcsZ* mutant did bind Calcofluor (fluorescence brightener 28) to a lower extent as the wild type (Fig. 2b; Additional file 3A) suggesting lower cellulose production.

Again unexpectedly, overexpression of BcsZ in the *bcsZ* mutant enhanced Congo red binding and the rdar morphotype even further (Fig. 2a). However, the Calcofluor binding phenotype was complemented (Fig. 2b; Additional file 3A). Rdar morphotype development and Calcofluor binding upon overexpression of BcsZ_E56A_ in the *bcsZ* mutant of UMR1 were similar to wild type BcsZ (Fig. 2a, b) indicating a role of BcsZ beyond catalytic activity.

To get detailed insights, we observed cellulose production within the colony by fluorescence microscopy. Cells from agar-grown colonies were carefully resuspended to maintain the aggregative structure, stained with Calcofluor and observed under the microscope (Fig. 2c). Cellulose production as judged by Calcofluor staining associated with clumps was readily observed in the wild type UMR1, in contrast to the *bcsA* deletion mutant, which showed residual clumping and staining due to the production of curli fimbriae. On average, *bcsZ* mutant clumps showed significantly higher Calcofluor staining than clumps of UMR1 wild type, which could be complemented by overexpression of BcsZ. Overexpression of the catalytic mutant BcsZ_E56A_ lead to a patchy distribution of the color stain in the clumps again indicating an effect of BcsZ beyond cellulose degradation (Fig. 2c).

**Fig. 3:**
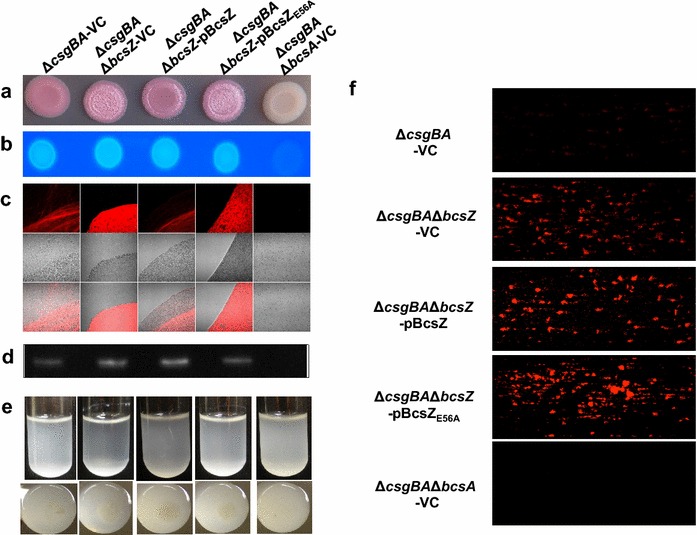
Biofilm phenotypes of the *bcsZ* deletion mutant of curli deficient *S.* Typhimurium derivatives of UMR1. **a** Pdar morphotype formation indicative for the expression of cellulose and **b** Calcoflour binding of *S.* Typhimurium UMR1Δ*csgBA* is enhanced upon deletion of *bcsZ*, but could be complemented by overexpression of BcsZ. **c** Calcofluor staining of cells resuspended from colonies grown on agar plates indicate higher cellulose production in the *bcsZ* mutant. Overexpression of BcsZ complemented the phenotype, while overexpression of the catalytic mutant BcsZ_E56A_ showed a patchy distribution of Calcofluor staining. **d** CsgD expression is not altered upon deletion and overexpression of *bcsZ*. **e** Cell clumping and biofilm formation of *S.* Typhimurium UMR1 Δ*csgBA* is enhanced upon deletion of *bcsZ* upon growth in M9 minimal medium for 16 h. Overexpression of BcsZ, but not of the catalytic mutant BcsZ_E56A_ complemented the phenotype. **f** Biofilm formation of UMR1 Δ*csgBA* and derivatives in microfluidic chambers. Enhanced biofilm formation was not complemented by BcsZ overexpression, and further enhanced by overexpression of the catalytic mutant BcsZ_E56A_. Sample order remains for **a**–**e** as shown on the *top panel*. *VC* pBAD30; *pBcsZ* BcsZ cloned in pBAD30; *pBcsZ*_*E56A*_ BcsZ catalytic mutant cloned in pBAD30

To monitor cellulose biosynthesis in the absence of the major extracellular matrix component curli fimbriae, which interferes with BcsZ dependent cellulose production (Fig. 1), we constructed the non-polar *bcsZ* mutant in the *csgBA* negative strain MAE14. The *bcsZ* mutant showed upregulated cellulose production as indicated by a more pronounced pdar morphotype (Fig. 3a). In accordance with a non-polar mutation, pdar morphotype expression was diminished by overexpression of BcsZ, but not the catalytically inactive BcsZ_E56A_ mutant. Changes in pdar morphotype were, though, not reflected by Calcofluor binding (Fig. 3b; Additional file 3B), suggesting that the amount of cellulose is altered upon differential expression of BcsZ. To this end, we also investigated cellulose production of MAE14 and derivatives by fluorescence microscopy (Fig. 3c). Calcofluor staining showed cellulose arranged in loose linear rows in the cell clusters of the wild type MAE14 as previously reported [9], while the *bcsA* mutant only showed single cells without Calcofluor staining. Deletion of *bcsZ* led to higher cellulose production and a network-like arrangement of the cellulose fibers, which could be complemented by overexpression of BcsZ. Overexpression of BcsZ_E56A_ led to a patchy pattern of Calcofluor staining again indicating a role of BcsZ beyond the catalytic activity (Fig. 3c).

As a third strain, we assessed cellulose production in MAE97 (Additional file 4). In MAE97, the *csgBA* gene is deleted and cellulose expressed constitutively at 28 °C and 37 °C, due to a mutation in the *csgD* promoter [38]. The MAE97Δ*bcsZ* showed upregulated cellulose production as characterized by a more pronounced pdar morphotype with deep pink color. In summary, *bcsZ* downregulates cellulose production. This effect is specific for the disruption of *bcsZ*, as the phenotype is complementable in the cellulose only background. In contrast, a polar mutant in *bcsZ* abolishes cellulose production equal to a *bcsC* mutant (Additional file 5; [9]). Second, as overexpression of BcsZ and BcsZ_E56A_ in the UMR1 wild type background did enhance instead of complement the upregulated rdar morphotype, BcsZ affects other pathways and cellulose biosynthesis in a complex fashion (Fig. 1c).

To this end, we tested the effect of *bcsZ* on the expression of the major biofilm regulator CsgD (Figs. 2d, 3d). Western blot analysis revealed that CsgD expression does not change consistently upon deletion and overexpression of BcsZ suggesting that the observed morphotype changes are independent of CsgD. Curli fimbriae, the other component contributing to rdar morphotype expression was not altered or even downregulated in the *bcsZ* mutant as well as upon overexpression of the BcsZ protein (Fig. 2d). In conclusion, changes in the rdar phenotype upon deletion and overexpression of BcsZ are not consistent with solely a glucosidase activity of BcsZ and potentially involve direct or indirect regulation of novel extracellular matrix component(s) in dependence of curli production. Also, to our knowledge, this is the first time an uncoupling of rdar morphotype development and CsgD expression is observed.

### *Expression of BcsZ in S.* Typhimurium *UMR1*

**Fig. 4:**
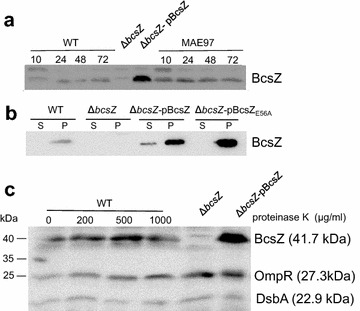
Expression and localization of BcsZ. **a** BcsZ is expressed throughout the growth phase of *S.* Typhimurium UMR1(WT) and MAE97. Expression of BcsZ was analyzed after growth on LB without salt agar plates from 10 to 72 h. **b** Analysis of BcsZ expression in the supernatant and cell pellet indicates BcsZ to be mainly cell associated. **c** BcsZ is not a surface associated protein. Treatment of the bacterial pellet with different concentrations of proteinase K does not alter the BcsZ signal at 41.7 kDa. Detection of cytoplasmic OmpR (27.3 kDa) and periplasmic DsbA (22.9 kDa) were cell integrity controls

Next, we monitored the expression of BcsZ in *S.* Typhimurium UMR1 using a polyclonal peptide antibody. BcsZ is expressed throughout the growth phase on agar plates whereby slightly diminished expression is observed at 10 h early growth (Fig. 4a). Bioinformatic analysis predicts BcsZ to be a periplasmic protein. Our experiments showed that BcsZ is cell associated and only a minor fraction of BcsZ is secreted into the supernatant upon overexpression (Fig. 4b). To assess whether BcsZ is present on the cell surface, we incubated the cells with proteinase K. Even high amounts of proteinase K did not degrade BcsZ indicating that BcsZ is not present on the cell surface, but most likely indeed is a periplasmic protein (Fig. 4c). This finding might also explain the weak cellulase phenotype on CMC-agar plates (Fig. 1b).

### Additional phenotypes affected by BcsZ

Cellulose biosynthesis is required for biofilm formation (adherence to solid surface) and cell–cell interaction (cell clumping) in liquid culture in M9 minimal medium at 28 °C [39]. UMR1, MAE14, MAE97 and their *bcsZ* derivatives were grown in M9 minimal medium. Consistently, *bcsZ* deletion mutants showed visibly higher cell aggregation complemented upon overexpression of BcsZ (Figs. 2e, 3e; Additional file 4B). BcsZ_E56A_ showed partial complementation, again indicating a potential role of the protein scaffold to phenotype expression. Enhanced clumping and cellulose production upon deletion of *bcsZ* was also observed with fluorescent microscopy (Additional file 6A, B). This phenotype was complemented by overexpression of BcsZ, but only partially by overexpression of BcsZ_E56A_. Higher biofilm formation upon deletion of *bcsZ* was not complemented (Fig. 3f).

Another phenotype associated with the rdar morphotype is pellicle formation in standing culture [30]. We measured pellicle strength by incremental applying weight (glass beads). The *bcsZ* mutant of UMR1 showed a more than sevenfold enhanced pellicle strength, which was complemented by wild type and also mutant BcsZ_E56A_ (Fig. 2f). Investigation of the *bcsZ bcsA* double mutant showed that pellicle formation is entirely dependent on the production of cellulose (Additional file 6C). As this is the most pronounced phenotype of a *bcsZ* mutant, BcsZ especially downregulates cellulose production in standing liquid culture.

**Fig. 5:**
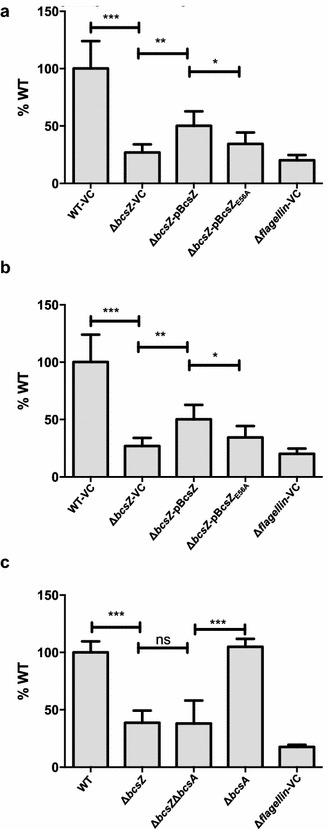
Swimming and swarming motility upon deletion of *bcsZ* in *S.* Typhimurium UMR1. **a** Swimming and **b** swarming motility of *S.* Typhimurium UMR1 (WT) was downregulated upon deletion of *bcsZ.* The phenotype cannot be complemented by overexpression of BcsZ or the BcsZ_E56A_ mutant. **c** The motility phenotype of a *bcsZ* mutant is not relieved upon deletion of *bcsA.* Plates were incubated at 37 °C. VC, *VC* pBAD30; *pBcsZ* BcsZ cloned in pBAD30; *pBcsZ*_*E56A*_ BcsZ catalytic mutant cloned in pBAD30; Δflagellin, negative control Δ*fliC* Δ*fljB*. *Bars* show the means of at least three independent experiments each in duplicate. *Error bar* indicates standard deviation (SD). ***p < 0.0005, **p<0.001, *p < 0.05; *ns* not significant using paired t-test

As an additional phenotype in *S.* Typhimurium, cellulose biosynthesis inhibits flagella based motility [36, 40]. Indeed, assessment of motility showed that swimming motility and, to a larger extent, swarming motility are downregulated in the *bcsZ* mutant of UMR1, which could only partially be complemented by overexpression of BcsZ (Fig. 5). Of note, this phenotype is also observed at 37 °C, a temperature where cellulose production on agar plates is not observed in the UMR1 wild type (Additional file 7). Deletion of *bcsA* in the *bcsZ* strain background, however, relieved the motility deficit only to a minor extent. We repeated the motility experiments in the MAE14 background (Additional file 8), a strain background where the pdar morphotype of the *bcsZ* mutant had been complemented (Fig. 3). As the motility defect of the *bcsZ* mutant was again hardly complemented deregulated expression of *bcsC* through the *tetA* promoter might be relevant (Additional file 5C).

### Role of BcsZ in host-pathogen interaction

**Fig. 6:**
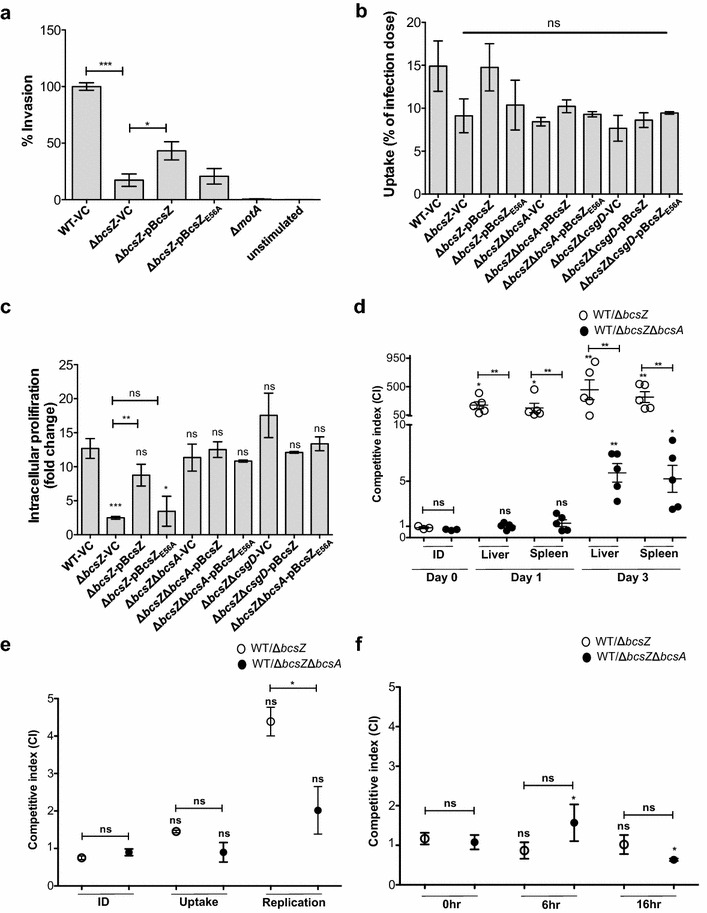
Host interaction phenotypes upon deletion of *bcsZ* in *S.* Typhimurium UMR1. **a** Invasion of epithelial cells by UMR1 (WT) and *bcsZ* mutant derivatives. **b** Uptake of UMR1 (WT) and *bcsZ* mutant derivatives in IFN-γ activated murine RAW264.7 macrophages at 2 h post infection (MOI of 10). For **a** and **b**, *error bar* indicates SD. **c** Intracellular proliferation (as fold change of uptake) of *bcsZ* mutant derivatives at 16 h post infection. *Error bar* indicates SD for two independent experiments, each in triplicates. For **a**–**c**, ***p < 0.0005, **p<0.001, *p < 0.05; *ns* not significant compared to WT-VC unless specified using paired t-test. **d** Competitive index (CI) of virulence of UMR1 (WT) against *bcsZ* mutant derivatives in organs of 6–8 week old female BALB/c mice (5 per group) on day 1 and 3 post oral infection. Each *circle* represents an individual mouse and *error bar* indicates SEM. Infection dose (ID) used for inoculation with a strain ratio of 1:1 for UMR1 (WT) and *bcsZ* mutant derivatives. Significance calculated for mean CI in organs at different time points compared to the inoculum and for the difference in CI for *bcsZ* mutant derivatives in the same organ at one time point. Difference between inocula is not statistically significant. **e** CI of fitness of UMR1 (WT) against *bcsZ* mutant derivatives for uptake (2 h) and proliferation (16 h) in IFN-gamma activated murine RAW264.7 macrophages (MOI of 10). All results are the means and *error bar* indicates SD for independent experiments, each in triplicates. Significance calculated for the average CI in uptake and proliferation compared to the inoculum and for the difference in CI for *bcsZ* mutant derivatives for uptake and proliferation. Difference between inocula is not statistically significant. *p < 0.05, *ns* not significant. **f** CI of fitness of UMR1 (WT) against *bcsZ* mutant derivatives in LB broth at 6 h and 16 h post inoculation. All results are the means and *error bar* indicates SD of two independent experiments, each in triplicates. Significance calculated for the average CI at different time points after inoculation compared to the inoculum and for the difference in CI for *bcsZ* mutant derivatives at different time points. Difference between inocula is not statistically significant. For **d**–**f**, ***p < 0.0005, **p<0.001, *p < 0.05; *ns* not significant using Kruskal–Wallis assessment with subsequent Dunn’s test to compare to inoculum and one-tailed unpaired t-test to compare two samples at the same time point

A motility phenotype for *bcsZ* was observed at 37 °C, although a *bcsZ* mutant does not affect cellulose production at 37 °C on agar plates (Additional file 8). This observation stimulated us to investigate the role of *bcsZ* in host-pathogen interaction. A key virulence phenotype of *S.* Typhimurium is invasion of epithelial cells [41, 42]. We investigated invasion of *S.* Typhimurium into the colon carcinoma epithelial cell line HT-29 (Fig. 6a). Of note, we could observe a 90 % reduction of invasion of the *bcsZ* mutant compared to the wild type UMR1, which was, again, only partially complemented by BcsZ overexpression.

Once having breached the epithelial barrier, *S.* Typhimurium can be taken up by host cells, such as macrophages, whereby it replicates in the host cells and is carried from the gut to the liver and spleen [41]. Investigation of the uptake of *S.* Typhimurium by macrophages showed a trend of the *bcsZ* mutant to be taken up with slightly less efficiency, 9 % of infection dose, compared to the wild type, 15 % of infection dose (Fig. 6b). Decreased wild type uptake can be restored by BcsZ overexpression, but not by mutant BcsZ_E56A_. Of note, uptake of the *S.* Typhimurium *bcsZ* mutant into macrophages could not be restored upon deletion of the cellulose synthase *bcsA* (Fig. 6b). Equally, deletion of the major biofilm regulator CsgD, previously demonstrated to contribute to the inhibition of epithelial cell line invasion at high c-di-GMP levels [14, 18], did not affect uptake of the *bcsZ* mutant (Fig. 6b).

In contrast to uptake, intracellular proliferation of *S.* Typhimurium in macrophages was severely affected by BcsZ. Indeed, the *bcsZ* mutant showed significantly lower proliferation than the wild type, a phenotype which could be restored by overexpression of BcsZ, but not its catalytic mutant BcsZ_E56A_ (Fig. 6c). Proliferation of the *bcsZ* mutant in the macrophages was restored upon individual deletion of the cellulose synthase BcsA and the major biofilm regulator CsgD in the *bcsZ* background. These findings indicate that enhanced CsgD dependent cellulose production contributes to diminished intracellular proliferation, but not to the uptake of the *bcsZ* mutant into macrophages (Fig. 6b, c).

As key virulence events are affected upon deletion of *bcsZ*, we also tested virulence of a *bcsZ* mutant in the mouse model for typhoid fever, a systemic infection model in *Salmonella* susceptible mice. Indeed, a competition assay against the wild type showed that the *bcsZ* mutant had a severe disadvantage, more than 50- and 100-fold, in liver and spleen on day 1 and day 3 of the infection, respectively (Fig. 6d). Deletion of the cellulose synthase BcsA in the *bcsZ* mutant background relieved the growth disadvantage and led to wild type growth in liver and spleen on day 1, while on day 3, the wild type still had a fivefold growth advantage. These results show that deregulation of cellulose production at body temperature is the major cause of the growth disadvantage of the *bcsZ* mutant in systemic infection.

To characterize the infection stage with a major growth disadvantage of the *bcsZ* mutant, we performed a competition assay for uptake and growth in macrophages (Fig. 6e). In concordance with the single infection experiments, the *bcsZ* mutant had a slight disadvantage in uptake, but a more severe, 4.5-fold disadvantage, in intracellular proliferation compared to wild type, which was relieved upon co-deletion of *bcsA*. In contrast, no growth advantage of the wild type was observed when the competition assay was performed in LB medium at 37 °C (Fig. 6f). These data indicate that proliferation in macrophages is one, but not the only phenotype which contributes to the growth advantage of the wild type against the *bcsZ* mutant in vivo.

## Discussion

Little is known about the regulation of cellulose biosynthesis. In this work, we show cellulose biosynthesis to be negatively regulated by the cellulase BcsZ encoded by the cellulose biosynthesis operon *bcsABZC* on the post-transcriptional level. BcsZ which belongs to the glycoside hydrolase family 8 (GH8) phenotypically reduces cellulose biosynthesis therefore this enzyme functions as an endoglucanase in vivo as previously observed in vitro [27]. Although glucanase activity has most commonly been reported for β-1,4-glucanases, a cellulase belonging to family 5 of glycoside hydrolase has been described to exhibit transglucosylase activity, consequently being involved in cellulose biosynthesis (Additional file 9; [43]). Indeed, such an enzyme should promote cellulose biosynthesis at least under distinct environmental conditions in vivo. Interestingly, cellulose biosynthesis promoting activity by the cellulase has been described for *K. xylinus*. In this bacterium the GH8 cellulase adjacent to the cellulose biosynthesis operon is secreted into the medium, but stays associated with the outer membrane to trigger cellulose biosynthesis [44]. Interestingly, plants also contain a cellulase (Korrigan) that is required for cellulose biosynthesis [45, 46]. The multifactorial role of the BcsZ cellulase on cellulose biosynthesis in the background of strains with and without co-expression of curli fimbriae as observed in this study might be partly related to the observations described above. In *Listeria monocytogenes*, a glucanase is required for exopolysaccharide synthesis, but leads to dissolution of exopolysaccharide-mediated clumps when added externally [47].

Cellulose biosynthesis is widespread in the bacterial kingdom. A GH8 family cellulase is an integral part of the type II cellulose biosynthesis operon and also found integral or adjacent to other classes of cellulose biosynthesis operons [25]. Assessment of phylogenetic relationship showed that cellulases encoded by the same type of cellulose biosynthesis operon grossly cluster together in the phylogenetic tree of GH8 cellulases (Additional file 10). In the plant symbiont *Rhizobium leguminosarum* bv. *trifolii*, reduction of cellulose biosynthesis upon cellulase expression was observed [48]. As the cellulase in *R. leguminosarum* bv. *trifolii* is more closely related with the cellulase of *K. xylinum* than *S.* Typhimurium, the phylogenetic relatedness does not seem to correlate directly with functionality.

Cellulases secreted by bacterial and fungal microorganisms mainly degrade plant cellulose and therefore play a major ecological role in global carbon cycling. These cellulases belong to various glycoside hydrolase families and occur e.g. in 32 % of the bacterial genomes [49]. For substrate recognition, these enzymes are usually modular and contain one or several carbohydrate binding domains. A carbohydrate binding domain is missing in cellulases encoded by or associated with cellulose biosynthesis operons. Therefore it remains an open question how cellulases associated with cellulose biosynthesis operons recognize their substrate.

Although cellulose biosynthesis operons are considered to be transcribed constitutively [9, 28], in the plant pathogen *Dickeya dadantii*, the transcription factor Fis negatively regulates expression of the cellulose biosynthesis operon [50]. However, the cellulose macromolecule is not constitutively produced, but activated post-transcriptionally by the ubiquitous secondary messenger c-di-GMP [26, 29]. Regulation of cellulose production by the cellulase BcsZ is a second post-translational mechanism to regulate cellulose biosynthesis.

Interestingly, complementation of the *bcsZ* mutant was only partial or inconsistent concerning the phenotypes rdar morphotype expression, motility, invasion of epithelial cells and uptake into macrophages. Whether enhanced expression of downstream BcsC in the non-polar deletion mutant (Additional file 5) or the biological function of the BcsZ scaffold overrides the complementation phenotype needs to be investigated in further studies.

A pronounced in vivo virulence phenotype upon deletion of the cellulase BcsZ was observed for *S*. Typhimurium with severe attenuation to colonize liver and spleen during competition experiments in mice (Fig. 6d). Virulence promotion of BcsZ was further seen in at least three ex vivo virulence phenotypes, promotion of invasion of epithelial cells, proliferation in macrophages and, to a minor extent, uptake by macrophages. Cumulatively, BcsZ affects virulence primarily by regulating cellulose biosynthesis in vivo at body temperature. Our results are consistent with deregulated cellulose production as an anti-virulence factor of acute infection to attenuate in vivo virulence and proliferation in macrophages [17]. In this context, it is worth to mention that in the plant symbiont *R. leguminosarum* bv. *trifolii*, the cellulase has a function independent of cellulose biosynthesis in the degradation of the non-crystalline root hair cell wall to establish symbiotic infection of the nitrogen-fixing bacterium [48, 51].

## Conclusions

In summary, we showed in this work that BcsZ diminishes cellulose production in *S.* Typhimurium to reduce biofilm formation and enhance virulence. Our results also indicate that the role of BcsZ goes beyond catalysis, whereby the molecular mechanism of catalysis-independent cellulase functionality needs to be further investigated.

## Additional files

**Additional file 1.** Strains and plasmids used in this study.

**Additional file 2.** Primers used in this study.

**Additional file 3.** **A** Quantification of cellulose production in *S.* Typhimurium UMR1 (WT) and **B** MAE14 (Δ*csgBA*) and derivatives. Quantification of Calcofluor binding showed that the *bcsZ* mutant of UMR1 bound less Calcofluor than the wild type. A representative experiment with eight technical replicates is shown. Error bars represent SEM. ***p< 0.0005, **p<0.001, *p<0.05; ns=not significant using Student’s paired t-test. VC= pBAD30; pBcsZ=*bcsZ* cloned in pBAD30. pBcsZE56A=catalytic mutant of BcsZ cloned in pBAD30; Δ*bcsA*, negative control; MAE97, positive control.

**Additional file 4.** **A** Pdar morphotype on agar plates and **B** cell clumping and biofilm formation in M9 medium of *S. typhimurium* MAE97 upon deletion of *bcsZ*. Samples: 1, MAE97 VC; 2, MAE97Δ*bcsZ* VC, 3, MAE97Δ*bcsZ* pBcsZ. VC, vector control; pBcsZ, BcsZ cloned in pBAD30.

**Additional file 5.** Phenotypes of polar and non-polar *bcsZ* mutants. **A** A polar ∆*bcsZ*::Cm mutant in UMR1 and MAE14 shows downregulation of rdar/pdar morphotype formation. Strains were grown on Congo red agar plates for 72 h at 28 °C. Deletion strains of UMR1 ∆*bcsA* and UMR1∆*csgD* served as negative controls. Strains: 1= UMR1, 2= UMR1 ∆*bcsZ*::Cm, 3= MAE14 (UMR1 Δ*csgBA*), 4= MAE14 ∆*bcsZ*::Cm, 5= UMR1 ∆*bcsA*, 6= UMR1 ∆*csgD.* **B** A *bcsC::*MudJ mutant in UMR1 and MAE5 shows strong reduction of rdar/pdar morphotype formation. Strains were grown on Congo red agar plates for 72h at 28°C. UMR1 ∆*bcsA* and UMR1 ∆*csgD* served as negative controls. Strains: 1= UMR1, 2= UMR1 *bcsC::*MudJ, 3= MAE5 (UMR1 Δ*csgA*), 4= MAE5 *bcsC::*MudJ, 5= UMR1 ∆*bcsA*, 6= UMR1 ∆*csgD.* **C** Upregulated BcsC expression levels in the non-polar ∆*bcsZ:tetRA* deletion background. Strains were grown on LB without salt plates at 28 °C for 16 h. Signals were detected with an anti 3xFLAG-antibody.

**Additional file 6.** **A** Cellulose expression and cell clumping of *S.* Typhimurium UMR1 (WT) and **B** *S.* Typhimurium MAE14 (UMR1Δ*csgBA*) is enhanced upon deletion of *bcsZ* during growth in M9 minimal medium for 16 h. Overexpression of BcsZ complemented the phenotype, while the catalytic mutant BcsZ_E56A_ showed an enhanced clumping phenotype. Samples: 1, UMR1 (A)/MAE14 (B) VC; 2, Δ*bcsZ* VC; 3, Δ*bcsZ* pBcsZ; 4, Δ*bcsZ* pBcsZ_E56A;_ 5, Δ*bcsA* VC. VC= pBAD30; pBcsZ=BcsZ cloned in pBAD30. pBcsZ_E56A_ =BcsZ catalytic mutant cloned in pBAD30. Δ*bcsA*, negative control. C. Pellicle strength of *S.* Typhimurium in standing culture enhanced upon deletion of *bcsZ* is entirely dependent on the cellulose synthase BcsA. *S*. Typhimurium UMR1 (WT) and derivatives were grown in LB without salt standing culture for 48 h at 28°C. Shown is a representative experiment with n=4 technical replicates. Error bars represent SEM. ***=p< 0.0005, **=p<0.001, *= p<0.05; ns=not significant using Student’s paired t-test. VC= pBAD30; pBcsZ= *bcsZ* cloned in pBAD30. Δ*bcsA* and Δ*bcsA* Δ*bcsZ*, negative controls.

**Additional file 7.** Colony morphotype of the *bcsZ* deletion mutant of *S.* Typhimurium MAE14 (UMR1 *ΔcsgBA*), the curli deficient *S.* Typhimurium derivative of UMR1. No alteration in the morphotype could be observed upon *bcsZ* deletion or overexpression after growth on Congo Red agar plates incubated at 37°C for 72 h. VC=pBAD30; pBcsZ=*bcsZ* cloned in pBAD30; pBcsZ_E56A_=catalytic mutant of BcsZ cloned in pBAD30.

**Additional file 8.** Swimming and swarming motility upon deletion of *bcsZ* in *S.* Typhimurium MAE14(UMR1 *ΔcsgBA*). **A** and **B** Swimming and C. and D. swarming motility of *S.* Typhimurium MAE14 was downregulated upon deletion of *bcsZ.* The phenotype cannot be complemented by overexpression of BcsZ or the *BcsZ*_E56A_ mutant. Plates were incubated at 28° (A and C) and 37°C (B and D). VC= pBAD30; pBcsZ= *bcsZ* cloned in pBAD30. Δf*lhDC*, negative control. Bars show the means of two independent experiments each in triplicates and error bar indicates standard deviation. ***=p< 0.0005, **=p<0.001, *= p<0.05; ns=not significant using Student’s paired t-test.

**Additional file 9.** Various functions of secreted and periplasmic cellulases in the degradation and synthesis of cellulose. Cellulases can belong to different families of glycoside hydrolases.

**Additional file 10.** Phylogenetic tree of glycoside hydrolase family 8 cellulases. BcsZ of *S.* Typhimurium is most closely related to other cellulases associated with cellulose biosynthesis operon class II. Characterized glycoside hydrolase family 8 cellulases are encoded by cellulose synthesizing bacteria with bacterial cellulose synthesis (BCS) operons of class I to III, but also by species not known to produce cellulose such as *Clostridium cellolyticum* and *Fibrobacter succinogenes.* GUN_BACCI: Q93HV0, *Bacillus circulans*; BcsZ_AQUAE: AAC07361, *Aquifex aeolicus* VF5; GUN_BACSP: P29019, *Bacillus* sp. KSM-330; GUN_CELUD: P18336, *Cellulomonas uda*; GUNC_CLOCE: P37699, *Clostridium cellulolyticum* ATCC 35319/DSM 5812; GUN_CLOJO: D1MX94, *Clostridium josui*; GUNY_DICD: P27032, *Dickeya dadantii* strain 3937; GUN_FIBSS: A7UG68, *Fibrobacter succinogenes* strain ATCC 19169/S85; GUNA_COMH: P37696, *Komagataeibacter hansenii*; GUN_JEONA: E2G4E3, *Jeongeupia naejangsanensis*; BcsZ1_KLEPN: YP_005229338, *Klebsiella pneumoniae* HS11286; BcsZ2_KLEPN: YP_005229346, *Klebsiella pneumoniae* HS11286; GUN_RHILT: Q83XK5, *Rhizobium leguminosarum* bv. Trifolii; GUN_CLOTH: *Clostridium thermocellum* strain ATCC 27405/DSM 1237; GUN ZYMMO: Q5NNK0, *Zymomonas mobilis* subsp. mobilis strain ATCC 31821 / ZM4; BcsZ Citkos: YP_001456454.1, *Citrobacter koseri* ATCC BAA-895; BcsZ_ECOLI: NP_756205.1, *Escherichia coli* CFT073; BcsZ_RAOOR: WP_015585558.1, *Raoultella ornithinolytica*; BcsZ_SERMA: WP_019452572.1, *Serratia marcescens*; BcsZ_PECCA: WP_012772817.1, *Pectobacterium carotovorum*; BcsZ_YEREN: WP_011817393.1, *Yersinia enterocolitica*; BcsZ_AERHY: AHE48194.1, *Aeromonas hydrophila* 4AK4; BcsZ_EDWTA: WP_005290796.1; *Edwardsiella tarda*; BcsZ_PSEPU: WP_012272816.1, *Pseudomonas putida*; BcsZ_VIBFI: WP_011263699, *Aliivibrio fisheri* ES114; BcsZ_BURMA: WP_011204605, *Burkholderia mallei* ATCC23344; BcsZ_CHRVI: WP_011136223, *Chromobacterium violaceum* ATCC12472; CelY_Kommed: WP_014106411, *Komagataeibacter medellinensis* NBRC3288; BcsZ_BORAV: CAJ50238, *Bordetella avium197N*; BcsZ_Metext: ABY29770, *Methylobacterium extorquens* PA1; BcsZ_Acicry: GAN74754, *Acidiphilium multivorum* AIU 301; BcsZ_Agrfab: NP_357300, *Agrobacterium fabrum* C5.8.

## References

[1] Römling U (2002). Molecular biology of cellulose production in bacteria. Res Microbiol.

[2] Barnhart DM, Su S, Baccaro BE, Banta LM, Farrand SK (2013). CelR, an ortholog of the diguanylate cyclase PleD of *Caulobacter*, regulates cellulose synthesis in *Agrobacterium tumefaciens*. Appl Environ Microbiol.

[3] Kawano Y, Saotome T, Ochiai Y, Katayama M, Narikawa R, Ikeuchi M (2011). Cellulose accumulation and a cellulose synthase gene are responsible for cell aggregation in the cyanobacterium *Thermosynechococcus vulcanus* RKN. Plant Cell Physiol.

[4] Recouvreux DO, Carminatti CA, Pitlovanciv AK, Rambo CR, Porto LM, Antonio RV (2008). Cellulose biosynthesis by the beta-proteobacterium, C*hromobacterium violaceum*. Curr Microbiol.

[5] Bassis CM, Visick KL (2010). The cyclic-di-GMP phosphodiesterase BinA negatively regulates cellulose-containing biofilms in *Vibrio fischeri*. J Bacteriol.

[6] Ude S, Arnold DL, Moon CD, Timms-Wilson T, Spiers AJ (2006). Biofilm formation and cellulose expression among diverse environmental *Pseudomonas* isolates. Environ Microbiol.

[7] Nobles DR, Romanovicz DK, Brown RM (2001). Cellulose in cyanobacteria. Origin of vascular plant cellulose synthase?. Plant Physiol.

[8] Zogaj X, Bokranz W, Nimtz M, Römling U (2003). Production of cellulose and curli fimbriae by members of the family *Enterobacteriaceae* isolated from the human gastrointestinal tract. Infect Immun.

[9] Zogaj X, Nimtz M, Rohde M, Bokranz W, Römling U (2001). The multicellular morphotypes of *Salmonella typhimurium* and *Escherichia coli* produce cellulose as the second component of the extracellular matrix. Mol Microbiol.

[10] Bokranz W, Wang X, Tschäpe H, Römling U (2005). Expression of cellulose and curli fimbriae by *Escherichia coli* isolated from the gastrointestinal tract. J Med Microbiol.

[11] Grantcharova N, Peters V, Monteiro C, Zakikhany K, Römling U (2010). Bistable expression of CsgD in biofilm development of *Salmonella enterica* serovar Typhimurium. J Bacteriol.

[12] Solano C, Garcia B, Valle J, Berasain C, Ghigo JM, Gamazo C, Lasa I (2002). Genetic analysis of *Salmonella enteritidis* biofilm formation: critical role of cellulose. Mol Microbiol.

[13] Simm R, Ahmad I, Rhen M, Le Guyon S, Römling U (2014). Regulation of biofilm formation in *Salmonella enterica* serovar Typhimurium. Future Microbiol.

[14] Lamprokostopoulou A, Monteiro C, Rhen M, Römling U (2010). Cyclic di-GMP signaling controls virulence properties of *Salmonella enterica* serovar Typhimurium at the mucosal lining. Environ Microbiol.

[15] Saldana Z, Xicohtencatl-Cortes J, Avelino F, Phillips AD, Kaper JB, Puente JL, Giron JA (2009). Synergistic role of curli and cellulose in cell adherence and biofilm formation of attaching and effacing *Escherichia coli* and identification of Fis as a negative regulator of curli. Environ Microbiol.

[16] Monteiro C, Saxena I, Wang X, Kader A, Bokranz W, Simm R, Nobles D, Chromek M, Brauner A, Brown RM, Römling U (2009). Characterization of cellulose production in *Escherichia coli* Nissle 1917 and its biological consequences. Environ Microbiol.

[17] Pontes MH, Lee EJ, Choi J, Groisman EA (2015). Salmonella promotes virulence by repressing cellulose production. Proc Natl Acad Sci USA.

[18] Ahmad I, Lamprokostopoulou A, Le Guyon S, Streck E, Barthel M, Peters V, Hardt WD, Römling U (2011). Complex c-di-GMP signaling networks mediate transition between virulence properties and biofilm formation in *Salmonella enterica* serovar Typhimurium. PLoS One.

[19] Rodriguez-Navarro DN, Dardanelli MS, Ruiz-Sainz JE (2007). Attachment of bacteria to the roots of higher plants. FEMS Microbiol Lett.

[20] Perez-Mendoza D, Aragon IM, Prada-Ramirez HA, Romero-Jimenez L, Ramos C, Gallegos MT, Sanjuan J (2014). Responses to elevated c-di-GMP levels in mutualistic and pathogenic plant-interacting bacteria. PLoS One.

[21] Morgan JL, McNamara JT, Zimmer J (2014). Mechanism of activation of bacterial cellulose synthase by cyclic di-GMP. Nat Struct Mol Biol.

[22] Saxena IM, Kudlicka K, Okuda K, Brown RM (1994). Characterization of genes in the cellulose-synthesizing operon (*acs* operon) of *Acetobacter xylinum*: implications for cellulose crystallization. J Bacteriol.

[23] Morgan JL, Strumillo J, Zimmer J (2013). Crystallographic snapshot of cellulose synthesis and membrane translocation. Nature.

[24] Whitney JC, Howell PL (2013). Synthase-dependent exopolysaccharide secretion in Gram-negative bacteria. Trends Microbiol.

[25] Römling U, Galperin MY (2015). Bacterial cellulose biosynthesis: diversity of operons, subunits, products, and functions. Trends Microbiol.

[26] Fang X, Ahmad I, Blanka A, Schottkowski M, Cimdins A, Galperin MY, Römling U, Gomelsky M (2014). GIL, a new c-di-GMP-binding protein domain involved in regulation of cellulose synthesis in enterobacteria. Mol Microbiol.

[27] Mazur O, Zimmer J (2011). Apo- and cellopentaose-bound structures of the bacterial cellulose synthase subunit BcsZ. J Biol Chem.

[28] Saxena IM, Brown RM (1995). Identification of a second cellulose synthase gene (acsAII) in *Acetobacter xylinum*. J Bacteriol.

[29] Ryjenkov DA, Simm R, Römling U, Gomelsky M (2006). The PilZ domain is a receptor for the second messenger c-di-GMP: the PilZ domain protein YcgR controls motility in enterobacteria. J Biol Chem.

[30] Römling U, Rohde M, Olsen A, Normark S, Reinköster J (2000). AgfD, the checkpoint of multicellular and aggregative behaviour in *Salmonella typhimurium* regulates at least two independent pathways. Mol Microbiol.

[31] Datsenko KA, Wanner BL (2000). One-step inactivation of chromosomal genes in *Escherichia coli* K-12 using PCR products. Proc Natl Acad Sci USA.

[32] Uzzau S, Figueroa-Bossi N, Rubino S, Bossi L (2001). Epitope tagging of chromosomal genes in *Salmonella*. Proc Natl Acad Sci USA.

[33] Beuzon CR, Holden DW (2001). Use of mixed infections with *Salmonella* strains to study virulence genes and their interactions in vivo. Microbes Infect.

[34] Chakravortty D, Hansen-Wester I, Hensel M (2002). *Salmonella* pathogenicity island 2 mediates protection of intracellular *Salmonella* from reactive nitrogen intermediates. J Exp Med.

[35] Lombard V, Golaconda Ramulu H, Drula E, Coutinho PM, Henrissat B (2014). The carbohydrate-active enzymes database (CAZy) in 2013. Nucleic Acids Res.

[36] Zorraquino V, Garcia B, Latasa C, Echeverz M, Toledo-Arana A, Valle J, Lasa I, Solano C (2013). Coordinated cyclic-di-GMP repression of *Salmonella motility* through YcgR and cellulose. J Bacteriol.

[37] Gerstel U, Römling U (2001). Oxygen tension and nutrient starvation are major signals that regulate agfD promoter activity and expression of the multicellular morphotype in *Salmonella typhimurium*. Environ Microbiol.

[38] Römling U, Sierralta WD, Eriksson K, Normark S (1998). Multicellular and aggregative behaviour of *Salmonella typhimurium* strains is controlled by mutations in the agfD promoter. Mol Microbiol.

[39] Simm R, Fetherston JD, Kader A, Römling U, Perry RD (2005). Phenotypic convergence mediated by GGDEF-domain-containing proteins. J Bacteriol.

[40] Le Guyon S, Simm R, Rhen M, Römling U (2014). Dissecting the c-di-GMP signaling network regulating motility in *Salmonella enterica* serovar Typhimurium. Environ Microbiol.

[41] Muller AJ, Kaiser P, Dittmar KE, Weber TC, Haueter S, Endt K, Songhet P, Zellweger C, Kremer M, Fehling HJ, Hardt WD (2012). *Salmonella* gut invasion involves TTSS-2-dependent epithelial traversal, basolateral exit, and uptake by epithelium-sampling lamina propria phagocytes. Cell Host Microbe.

[42] Lorkowski M, Felipe-Lopez A, Danzer CA, Hansmeier N, Hensel M (2014). *Salmonella enterica* invasion of polarized epithelial cells is a highly cooperative effort. Infect Immun.

[43] Berlemont R, Delsaute M, Pipers D, D’Amico S, Feller G, Galleni M, Power P (2009). Insights into bacterial cellulose biosynthesis by functional metagenomics on Antarctic soil samples. ISME J.

[44] Nakai T, Sugano Y, Shoda M, Sakakibara H, Oiwa K, Tuzi S, Imai T, Sugiyama J, Takeuchi M, Yamauchi D, Mineyuki Y (2013). Formation of highly twisted ribbons in a carboxymethylcellulose gene-disrupted strain of a cellulose-producing bacterium. J Bacteriol.

[45] Peng L, Kawagoe Y, Hogan P, Delmer D (2002). Sitosterol-beta-glucoside as primer for cellulose synthesis in plants. Science.

[46] Mansoori N, Timmers J, Desprez T, Kamei CL, Dees DC, Vincken JP, Visser RG, Hofte H, Vernhettes S, Trindade LM (2014). KORRIGAN1 interacts specifically with integral components of the cellulose synthase machinery. PLoS One.

[47] Koseoglu VK, Heiss C, Azadi P, Topchiy E, Guvener ZT, Lehmann TE, Miller KW, Gomelsky M (2015). Listeria monocytogenes exopolysaccharide: origin, structure, biosynthetic machinery and c-di-GMP-dependent regulation. Mol Microbiol.

[48] Robledo M, Rivera L, Jimenez-Zurdo JI, Rivas R, Dazzo F, Velazquez E, Martinez-Molina E, Hirsch AM, Mateos PF (2012). Role of *Rhizobium* endoglucanase CelC2 in cellulose biosynthesis and biofilm formation on plant roots and abiotic surfaces. Microb Cell Fact.

[49] Berlemont R, Martiny AC (2013). Phylogenetic distribution of potential cellulases in bacteria. Appl Environ Microbiol.

[50] Prigent-Combaret C, Zghidi-Abouzid O, Effantin G, Lejeune P, Reverchon S, Nasser W (2012). The nucleoid-associated protein Fis directly modulates the synthesis of cellulose, an essential component of pellicle-biofilms in the phytopathogenic bacterium *Dickeya dadantii*. Mol Microbiol.

[51] Robledo M, Jimenez-Zurdo JI, Velazquez E, Trujillo ME, Zurdo-Pineiro JL, Ramirez-Bahena MH, Ramos B, Diaz-Minguez JM, Dazzo F, Martinez-Molina E, Mateos PF (2008). *Rhizobium* cellulase CelC2 is essential for primary symbiotic infection of legume host roots. Proc Natl Acad Sci USA.

