# Supplementary material for: BcsZ inhibits biofilm phenotypes and promotes virulence by blocking cellulose production in Salmonella enterica serovar Typhimurium
Source: Microb Cell Fact. 2016 Oct 19;15:177. doi: 10.1186/s12934-016-0576-6 (PMC5070118; doi:10.1186/s12934-016-0576-6)
Supplement: Supplementary file 1 — Additional file 1. Strains and plasmids used in this study. [file 12934_2016_576_MOESM1_ESM.pdf]

Additional file 1: Strains and plasmids used in this study.

Strain or plasmid	Description	Reference/ Source
***E. coli* K-12**		
DH5α	λφ80d*lacZ* ΔM15 Δ(*lacZYA-argF*)*U169* *recA1 endA1 hsdR17*(r_K^- m_K^-) *supE44 thi-1* *gyrA relA1*	New England Biolabs
TOP10	F- *mcrA* Δ(*mrr-hsdRMS-mcrBC*) φ80lacZΔM15 Δ*lacX74 nupG recA1* *araD139* Δ(*ara-leu*)7697 *galE15 galK16* *rpsL*(Str^R) *endA1* λ^-	Invitrogen
***S.* Typhimurium ATCC14028**		
UMR1	ATCC14028 Nal^r, $cellulose_{28°C}$, curli $fimbriae_{28°C}$	[1]
MAE5	UMR1 *ΔcsgA101*	[1]
MAE14	UMR1 Δ*csgBA101*::Km^r, $cellulose_{28°C}$	[2]
MAE50	UMR1 Δ*csgD101*	[2]
MAE97	UMR1 p*csgD1* Δ*csgBA102*, $cellulose_{28/37°C}$	[2]
MAE98	UMR1 Δ*bcsZ101*::*tetRA*	This study
MAE99	MAE14 Δ*bcsZ101*::*tetRA*	This study

MAE108	Δ*fliC*::Cm Δ*fljB*	[3]
MAE155	UMR1 p*csgD1* Δ*bcsC101:*:MudJ	[4]
MAE222	UMR1 Δ*bcsA101*::MudJ	[4]
MAE224	UMR1 Δ*bcsZ101*::Cm	This study
MAE1254	MAE97 Δ*bcsZ101*::*tetRA*	This study
MAE1263	MAE97 BcsZE56A	This study
MAE1267	UMR1 Δ*bcsA*::GT2PilZ::*tetRA*	This study
MAE1473	Δ*flhDC*::Cm	[5]
MAE1766	Δ*motA*::Cm	[5]
MAE1873	UMR1 *bcsC*-3xFLAG	This study
MAE1900	UMR1 Δ*csgD101* Δ*bcsZ101*::*tetRA*	This study
MAE1903	UMR1 Δ*bcsZ101*::*tetRA* Δ*bcsA101*::MudJ	This study
MAE2124	UMR1 Δ*bcsC101:*:MudJ	This study
MAE2125	UMR1 Δ*bcsZ101*::*tetRA* *bcsC*-3xFLAG	This study
MAE2126	MAE14 Δ*bcsZ101*::Cm	This study
MAE2127	MAE5 Δ*bcsC101:*:MudJ	This study
**Plasmids**		
pBAD30	pACYC184 ori; L-arabinose regulated *araC*	[6]

	P_{BAD} promoter; Amp^r	
pKD3	FRT-cat-FRT; Cm^r, Amp^r	[7]
pKD46	$P_{araB}\alpha\beta\gamma$; Amp^r	[7]
pSUB11	3xFLAG-Km^r	[8]
pSRKTc	pBBR1 ori, *lacI^q*, P*lacZ*, Tet^r	[9]
pACYC184	pACYC ori; Tet^r	New England Biolabs
pBcsZ	*bcsZ* cloned in *Sac*I/*Sph*I site in pBAD30 with a 8x His-Tag	This study
$pBcsZ_{E56A}$	$bcsZ_{E56A}$ cloned in *Sac*I/*Sph*I site in pBAD30 with a 8x His-Tag	This study

[1], American Type Culture Collection.

References

1. Römling, U., et al., *Curli fibers are highly conserved between Salmonella typhimurium and Escherichia coli with respect to operon structure and regulation.* J Bacteriol, 1998. **180**(3): p. 722-31.
2. Romling, U., et al., *AgfD, the checkpoint of multicellular and aggregative behaviour in Salmonella typhimurium regulates at least two independent pathways.* Mol Microbiol, 2000. **36**(1): p. 10-23.
3. Rochon, M. and U. Romling, *Flagellin in combination with curli fimbriae elicits an immune response in the gastrointestinal epithelial cell line HT-29.* Microbes Infect, 2006. **8**(8): p. 2027-33.
4. Zogaj, X., et al., *The multicellular morphotypes of Salmonella typhimurium and Escherichia coli produce cellulose as the second component of the extracellular matrix.* Mol Microbiol, 2001. **39**(6): p. 1452-63.

5. Ahmad, I., et al., *The EAL-like protein STM1697 regulates virulence phenotypes, motility and biofilm formation in Salmonella typhimurium.* Mol Microbiol, 2013. **90**(6): p. 1216-32.
6. Guzman, L.M., et al., *Tight regulation, modulation, and high-level expression by vectors containing the arabinose PBAD promoter.* J Bacteriol, 1995. **177**(14): p. 4121-30.
7. Datsenko, K.A. and B.L. Wanner, *One-step inactivation of chromosomal genes in Escherichia coli K-12 using PCR products.* Proc Natl Acad Sci U S A, 2000. **97**(12): p. 6640-5.
8. Uzzau, S., et al., *Epitope tagging of chromosomal genes in Salmonella.* Proc Natl Acad Sci U S A, 2001. **98**(26): p. 15264-9.
9. Khan, S.R., et al., *Broad-host-range expression vectors with tightly regulated promoters and their use to examine the influence of TraR and TraM expression on Ti plasmid quorum sensing.* Appl Environ Microbiol, 2008. **74**(16): p. 5053-62.
