# Supplementary material for: BcsZ inhibits biofilm phenotypes and promotes virulence by blocking cellulose production in Salmonella enterica serovar Typhimurium
Source: Microb Cell Fact. 2016 Oct 19;15:177. doi: 10.1186/s12934-016-0576-6 (PMC5070118; doi:10.1186/s12934-016-0576-6)
Supplement: Supplementary file 2 — Additional file 2. Primers used in this study. [file 12934_2016_576_MOESM2_ESM.pdf]

Additional file 2: Primers used in this study

Primer	Sequence
	Cloning of *bcsZ* into pBAD30
BcsZclon_N	ACAGCGCTCAAGGAGGGTAACATATGATGACTATGCTGCGCGGATGG
BcsZclon_C	CAGACGCATGCTTAATGGTGGTGATGATGGTGATGGTGACGTGAACTTACGCATTCCTGGCCCCGTCAG
	Site-directed mutagenesis $BcsZ_{E56A}$
BcsZNE56A	CAGGGACGCGTTATCGATCCGGGCGATGCGCGAAAAATTACCACCTCCGCAGGG
BcsZCE56A	CTGTCGCTGCACGGCCTGGGCGTCGCGATTTTGCAGGAAAGGCAGTAAGGC
	bcsZ flanking primers
BcsZup	TGCTGGCGGTACTGGCGGCCCTCAGTGTGG
BcsZdown	GTCGCTGCGCCGGCAAGCGGTGTTAGCG
	Construction of Δ*bcsZ::tetRA*
BcsZ-TetRA	CAGGGACGCGTTATCGATCCGGGCGATGCGCAAAAATTACCACCTCCTTAAGACCCACTTTCACATTTAAG
BcsZC-TetRA	CTGTCGCTGCACGGCCTGGGCGCGATTTTGCAGGAAAGGCAGTA

	ACTAAGCACTTGTCTCCTGTTTACTC
Construction of Δ*bcsZ::Cm*	
BcsZ-CmDel-Start	ATGACTATGCTGCGCGGATGGATAACGATGCTCGTCATGCGTGTAGGCTGGAGCTGCTTC
BcsZ-CmDel-Stop	TTAACGTGAACTTACGCATTCCTGGCCCCAGTCAGGTAATCATATGAATATCCTCCTTAG
Control primers Δ*bcsZ::Cm*	
Cm inside	CTCCAGAGCGATGAAAACGT
BcsZcontrol Rev	CGGCAAAAAAGCGTGCATGAGAC
Control primers *bcsC*::MudJ	
bcsC_200upstream	CCGGTCGGGTTTTCCGCCGCCTTACTGCCT
mudJ-5primerc	TTATCGTGAAACGCTTTCGCGTTTTTCGTG
Construction of *bcsC*-3XFLAG	
BcsC3XFLAG forw	GATTTACCGCCGCAGCCTCTGGTGCCTTACGCTGACTGGgactacaaagaccatgacgg
BcsC3XFLAG Rev	GCCTACGACGCCTGTCAATTCGTAGGCCTGACGCAATGAatatgaatatcctccttag

Control primers *bcsC*-3XFLAG	
bcsCintfw	CAGCGTACGGAAAACTGGTC
Km inside rv	GTTTCGCTTGGTGGTCGAAT
