# Supplementary figures and images for: BcsZ inhibits biofilm phenotypes and promotes virulence by blocking cellulose production in Salmonella enterica serovar Typhimurium
Source: Microb Cell Fact. 2016 Oct 19;15:177. doi: 10.1186/s12934-016-0576-6 (PMC5070118; doi:10.1186/s12934-016-0576-6)

**A**

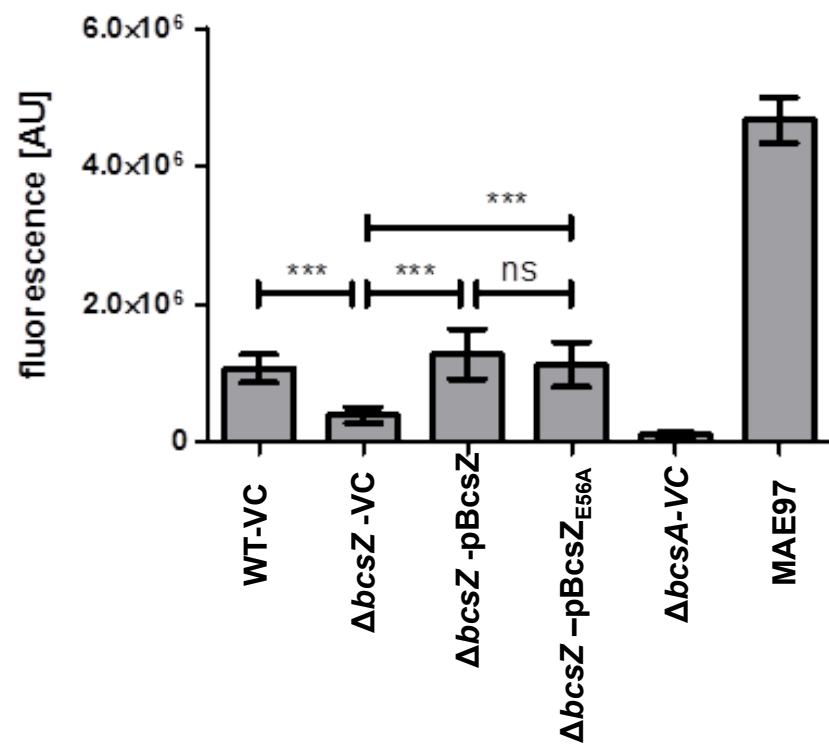

**B**

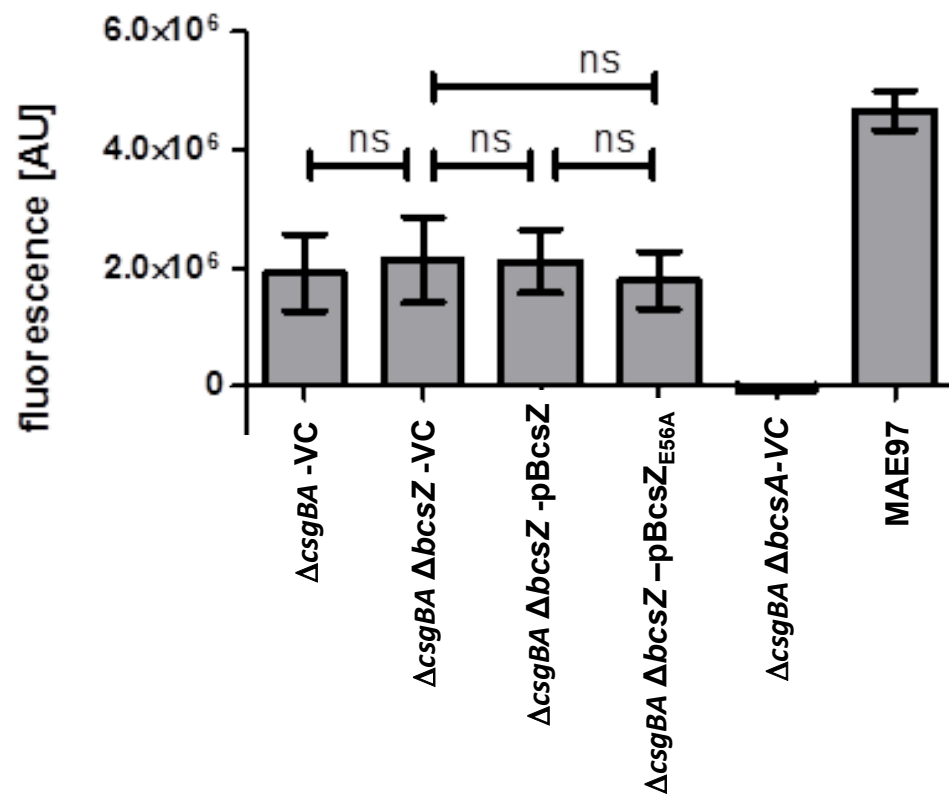

Supplement: Supplementary file 3 — Additional file 3. A Quantification of cellulose production in S. Typhimurium UMR1 (WT) and B MAE14 (ΔcsgBA) and derivatives. Quantification of Calcofluor binding showed that the bcsZ mutant of UMR1 bound less Calcofluor than the wild type. A representative experiment with eight technical replicates is shown. Error bars represent SEM. ***p< 0.0005, **p<0.001, *p<0.05; ns=not significant using Student’s paired t-test. VC= pBAD30; pBcsZ=bcsZ cloned in pBAD30. pBcsZE56A=catalytic mutant of BcsZ cloned in pBAD30; ΔbcsA, negative control; MAE97, positive control. [file 12934_2016_576_MOESM3_ESM.pdf]

# Additional file 4

MAE97-VC

MAE97 $\Delta$ bcsZ-VC

MAE97 $\Delta$ bcsZ-pBcsZ

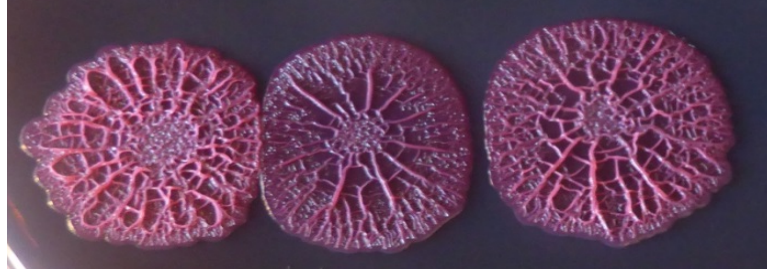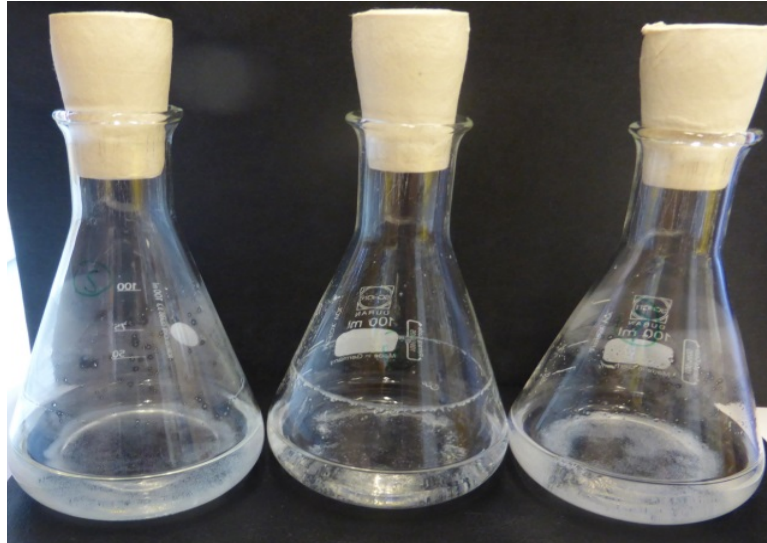

Supplement: Supplementary file 4 — Additional file 4. A Pdar morphotype on agar plates and B cell clumping and biofilm formation in M9 medium of S. typhimurium MAE97 upon deletion of bcsZ. Samples: 1, MAE97 VC; 2, MAE97ΔbcsZ VC, 3, MAE97ΔbcsZ pBcsZ. VC, vector control; pBcsZ, BcsZ cloned in pBAD30. [file 12934_2016_576_MOESM4_ESM.pdf]

# Additional file 5

**A**

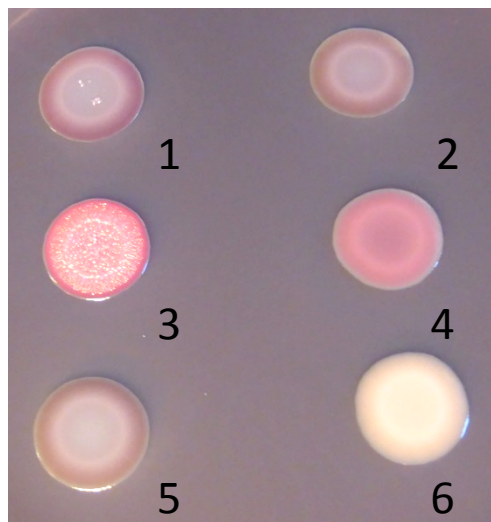

**B**

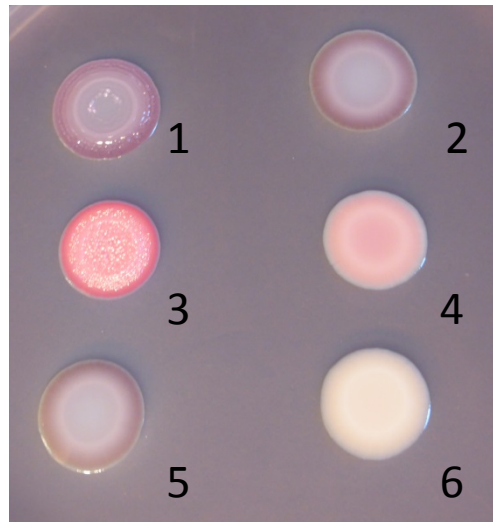

**C**

UMR1

UMR1  
*bcsC3xFLAG*

UMR1 $\Delta$ *bcsZ:tetRA*  
*bcsC3xFLAG*

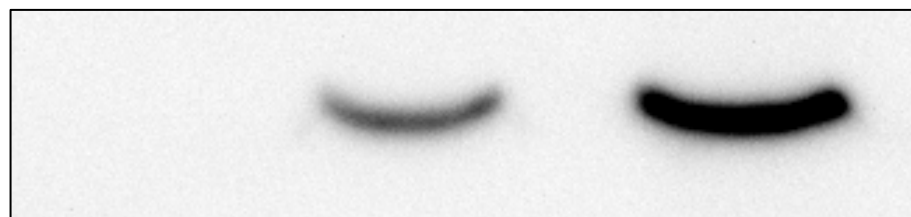

$\alpha$ -FLAG

Supplement: Supplementary file 5 — Additional file 5. Phenotypes of polar and non-polar bcsZ mutants. A A polar ∆bcsZ::Cm mutant in UMR1 and MAE14 shows downregulation of rdar/pdar morphotype formation. Strains were grown on Congo red agar plates for 72 h at 28 °C. Deletion strains of UMR1 ∆bcsA and UMR1∆csgD served as negative controls. Strains: 1= UMR1, 2= UMR1 ∆bcsZ::Cm, 3= MAE14 (UMR1 ΔcsgBA), 4= MAE14 ∆bcsZ::Cm, 5= UMR1 ∆bcsA, 6= UMR1 ∆csgD. B A bcsC::MudJ mutant in UMR1 and MAE5 shows strong reduction of rdar/pdar morphotype formation. Strains were grown on Congo red agar plates for 72h at 28°C. UMR1 ∆bcsA and UMR1 ∆csgD served as negative controls. Strains: 1= UMR1, 2= UMR1 bcsC::MudJ, 3= MAE5 (UMR1 ΔcsgA), 4= MAE5 bcsC::MudJ, 5= UMR1 ∆bcsA, 6= UMR1 ∆csgD. C Upregulated BcsC expression levels in the non-polar ∆bcsZ:tetRA deletion background. Strains were grown on LB without salt plates at 28 °C for 16 h. Signals were detected with an anti 3xFLAG-antibody. [file 12934_2016_576_MOESM5_ESM.pdf]

# Additional file 6

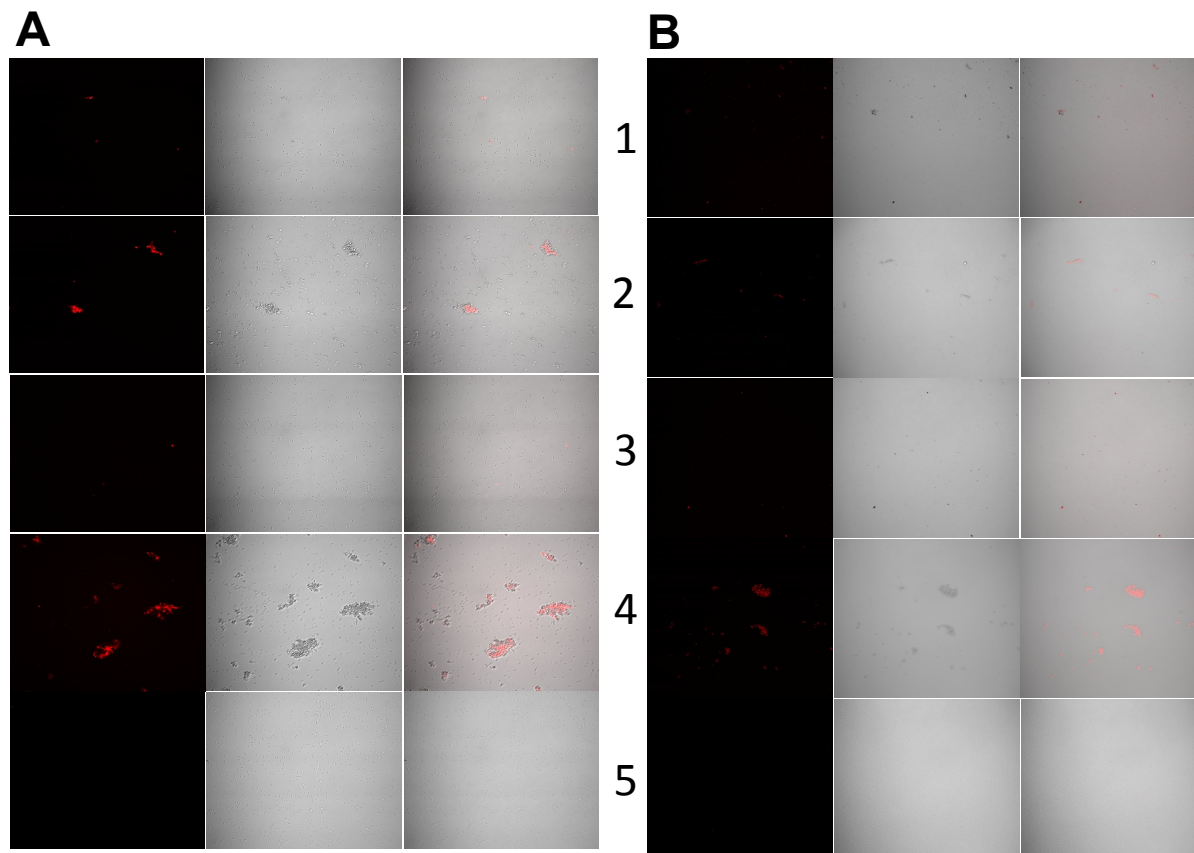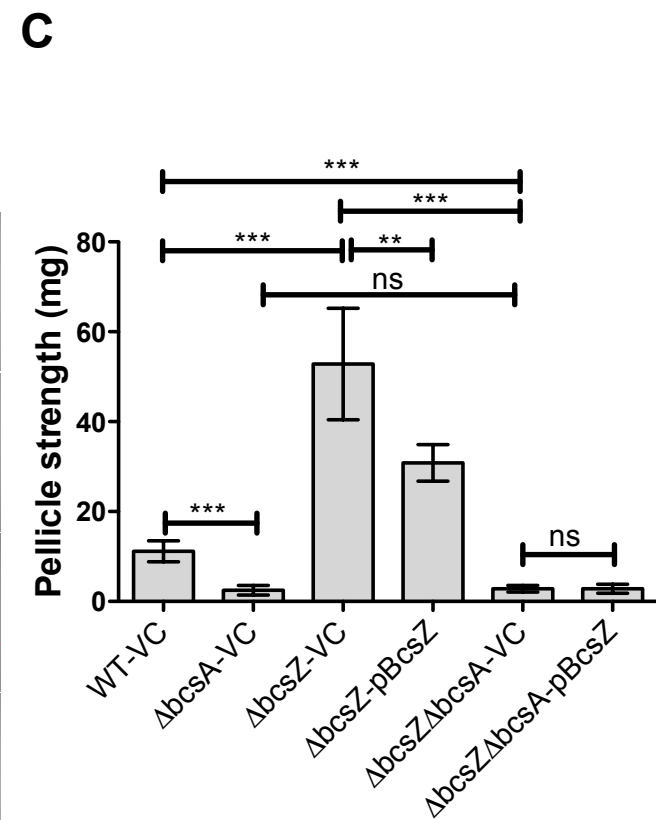

Supplement: Supplementary file 6 — Additional file 6. A Cellulose expression and cell clumping of S. Typhimurium UMR1 (WT) and B S. Typhimurium MAE14 (UMR1ΔcsgBA) is enhanced upon deletion of bcsZ during growth in M9 minimal medium for 16 h. Overexpression of BcsZ complemented the phenotype, while the catalytic mutant BcsZE56A showed an enhanced clumping phenotype. Samples: 1, UMR1 (A)/MAE14 (B) VC; 2, ΔbcsZ VC; 3, ΔbcsZ pBcsZ; 4, ΔbcsZ pBcsZE56A; 5, ΔbcsA VC. VC= pBAD30; pBcsZ=BcsZ cloned in pBAD30. pBcsZE56A =BcsZ catalytic mutant cloned in pBAD30. ΔbcsA, negative control. C. Pellicle strength of S. Typhimurium in standing culture enhanced upon deletion of bcsZ is entirely dependent on the cellulose synthase BcsA. S. Typhimurium UMR1 (WT) and derivatives were grown in LB without salt standing culture for 48 h at 28°C. Shown is a representative experiment with n=4 technical replicates. Error bars represent SEM. ***=p< 0.0005, **=p<0.001, *= p<0.05; ns=not significant using Student’s paired t-test. VC= pBAD30; pBcsZ= bcsZ cloned in pBAD30. ΔbcsA and ΔbcsA ΔbcsZ, negative controls. [file 12934_2016_576_MOESM6_ESM.pdf]

# Additional file 8

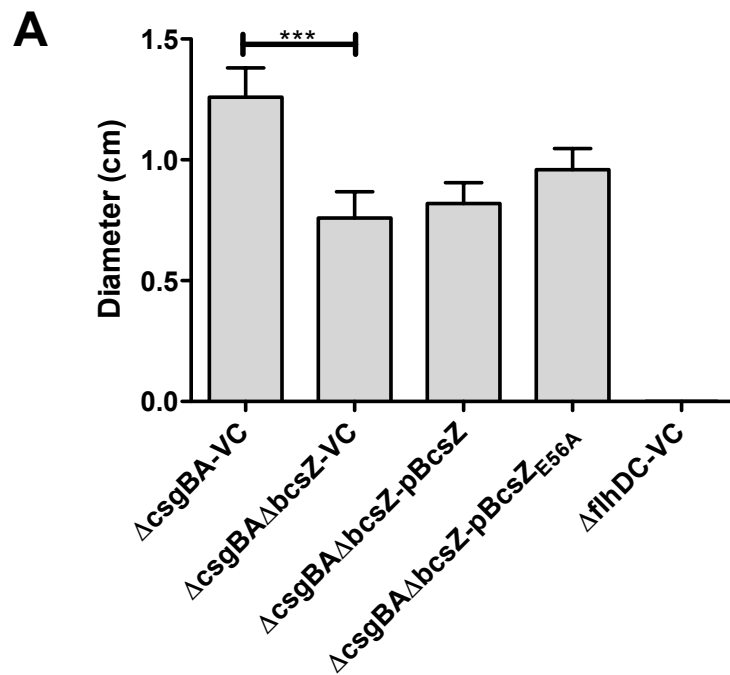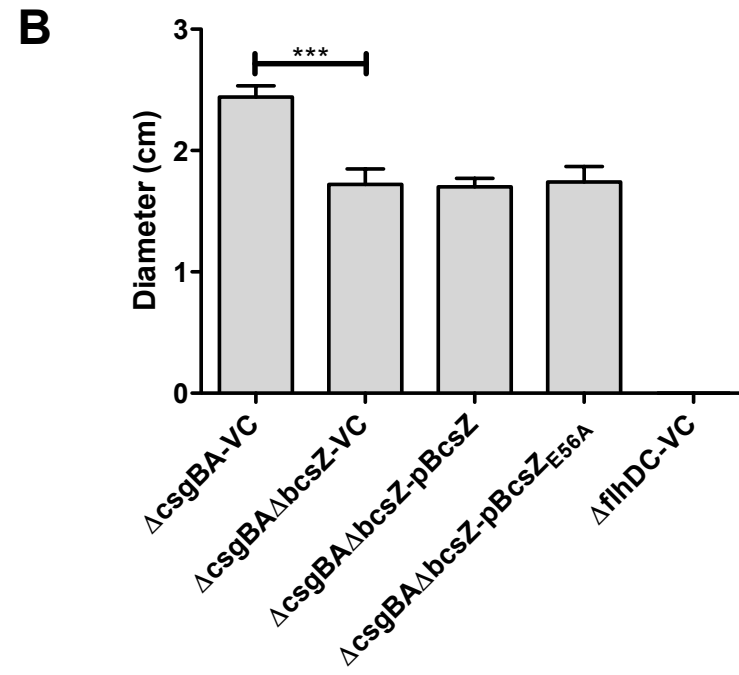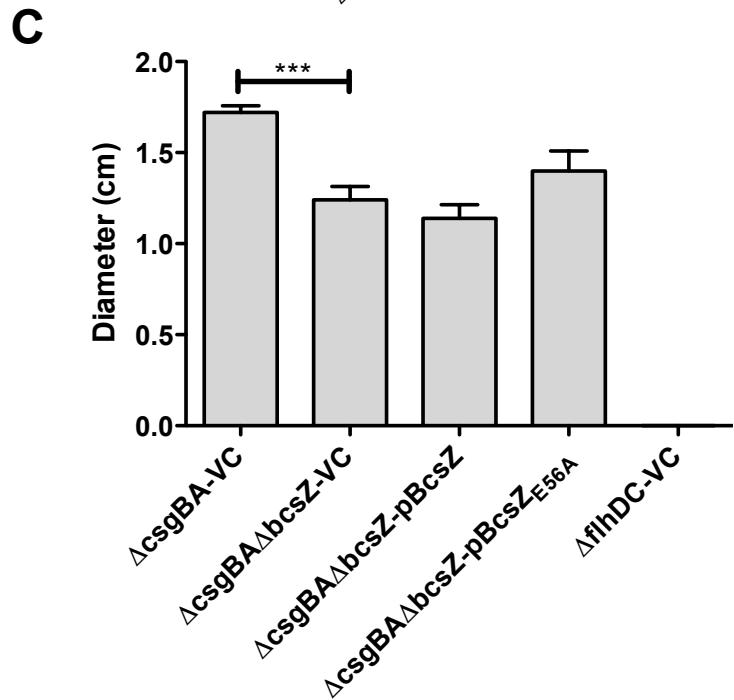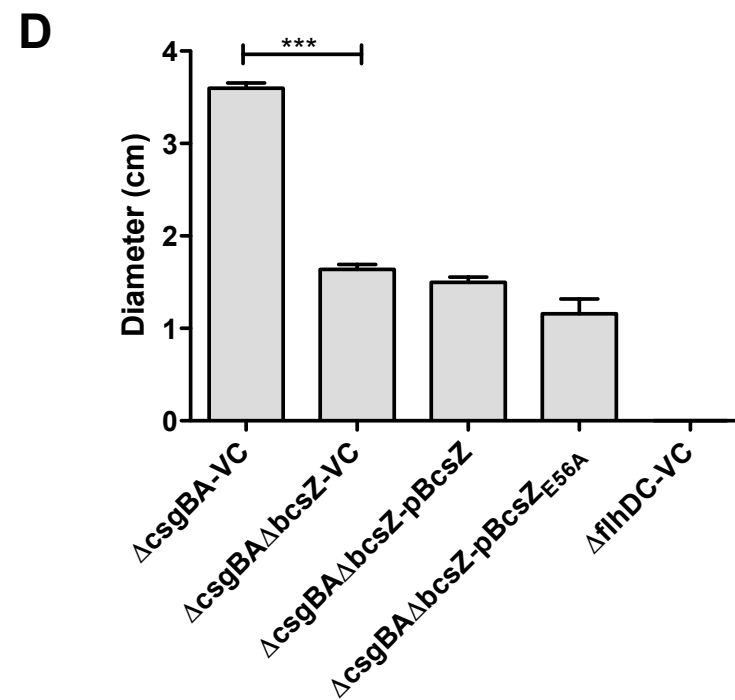

Supplement: Supplementary file 8 — Additional file 8. Swimming and swarming motility upon deletion of bcsZ in S. Typhimurium MAE14(UMR1 ΔcsgBA). A and B Swimming and C. and D. swarming motility of S. Typhimurium MAE14 was downregulated upon deletion of bcsZ. The phenotype cannot be complemented by overexpression of BcsZ or the BcsZ E56A mutant. Plates were incubated at 28° (A and C) and 37°C (B and D). VC= pBAD30; pBcsZ= bcsZ cloned in pBAD30. ΔflhDC, negative control. Bars show the means of two independent experiments each in triplicates and error bar indicates standard deviation. ***=p< 0.0005, **=p<0.001, *= p<0.05; ns=not significant using Student’s paired t-test. [file 12934_2016_576_MOESM8_ESM.pdf]

## Additional file 9

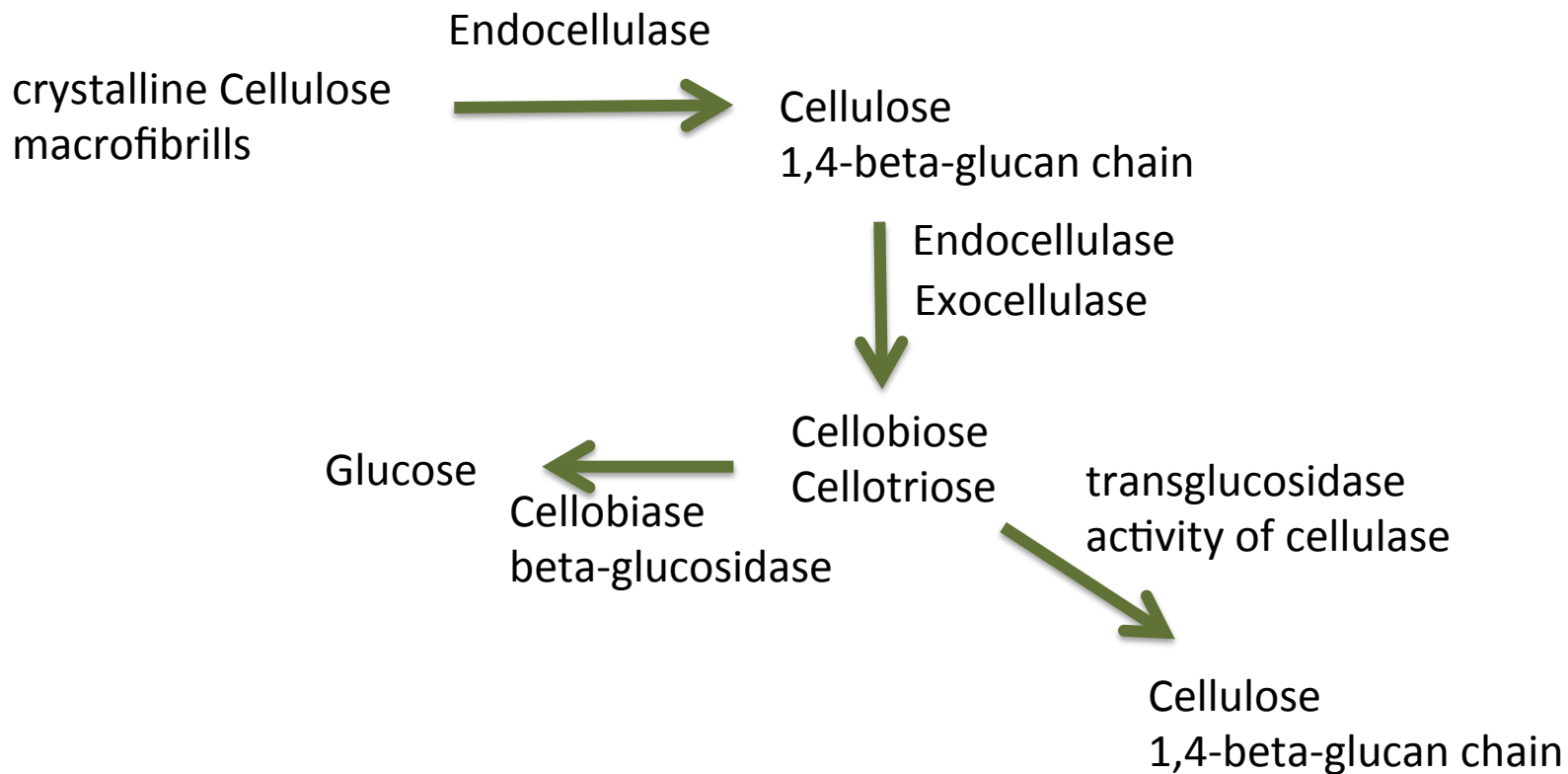

Supplement: Supplementary file 9 — Additional file 9. Various functions of secreted and periplasmic cellulases in the degradation and synthesis of cellulose. Cellulases can belong to different families of glycoside hydrolases. [file 12934_2016_576_MOESM9_ESM.pdf]

Additional  
file 10

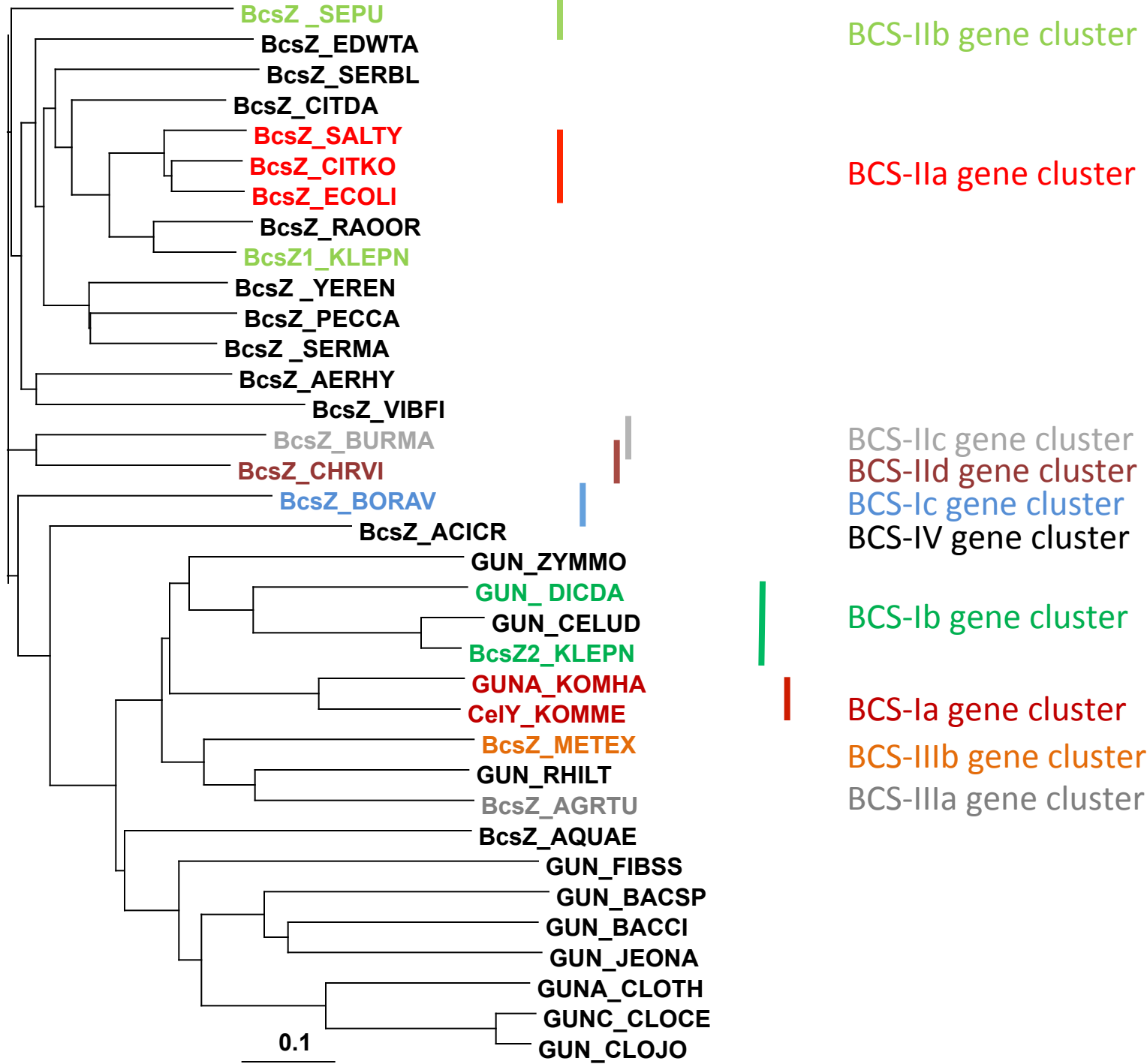

Supplement: Supplementary file 10 — Additional file 10. Phylogenetic tree of glycoside hydrolase family 8 cellulases. BcsZ of S. Typhimurium is most closely related to other cellulases associated with cellulose biosynthesis operon class II. Characterized glycoside hydrolase family 8 cellulases are encoded by cellulose synthesizing bacteria with bacterial cellulose synthesis (BCS) operons of class I to III, but also by species not known to produce cellulose such as Clostridium cellolyticum and Fibrobacter succinogenes. GUN_BACCI: Q93HV0, Bacillus circulans; BcsZ_AQUAE: AAC07361, Aquifex aeolicus VF5; GUN_BACSP: P29019, Bacillus sp. KSM-330; GUN_CELUD: P18336, Cellulomonas uda; GUNC_CLOCE: P37699, Clostridium cellulolyticum ATCC 35319/DSM 5812; GUN_CLOJO: D1MX94, Clostridium josui; GUNY_DICD: P27032, Dickeya dadantii strain 3937; GUN_FIBSS: A7UG68, Fibrobacter succinogenes strain ATCC 19169/S85; GUNA_COMH: P37696, Komagataeibacter hansenii; GUN_JEONA: E2G4E3, Jeongeupia naejangsanensis; BcsZ1_KLEPN: YP_005229338, Klebsiella pneumoniae HS11286; BcsZ2_KLEPN: YP_005229346, Klebsiella pneumoniae HS11286; GUN_RHILT: Q83XK5, Rhizobium leguminosarum bv. Trifolii; GUN_CLOTH: Clostridium thermocellum strain ATCC 27405/DSM 1237; GUN ZYMMO: Q5NNK0, Zymomonas mobilis subsp. mobilis strain ATCC 31821 / ZM4; BcsZ Citkos: YP_001456454.1, Citrobacter koseri ATCC BAA-895; BcsZ_ECOLI: NP_756205.1, Escherichia coli CFT073; BcsZ_RAOOR: WP_015585558.1, Raoultella ornithinolytica; BcsZ_SERMA: WP_019452572.1, Serratia marcescens; BcsZ_PECCA: WP_012772817.1, Pectobacterium carotovorum; BcsZ_YEREN: WP_011817393.1, Yersinia enterocolitica; BcsZ_AERHY: AHE48194.1, Aeromonas hydrophila 4AK4; BcsZ_EDWTA: WP_005290796.1; Edwardsiella tarda; BcsZ_PSEPU: WP_012272816.1, Pseudomonas putida; BcsZ_VIBFI: WP_011263699, Aliivibrio fisheri ES114; BcsZ_BURMA: WP_011204605, Burkholderia mallei ATCC23344; BcsZ_CHRVI: WP_011136223, Chromobacterium violaceum ATCC12472; CelY_Kommed: WP_014106411, Komagataeibacter medellinensis NBRC3288; BcsZ_BORA [file 12934_2016_576_MOESM10_ESM.pdf]
