# Supplementary material for: BcsZ inhibits biofilm phenotypes and promotes virulence by blocking cellulose production in Salmonella enterica serovar Typhimurium
Source: Microb Cell Fact. 2016 Oct 19;15:177. doi: 10.1186/s12934-016-0576-6 (PMC5070118; doi:10.1186/s12934-016-0576-6)
Supplement: Supplementary file 7 — Additional file 7. Colony morphotype of the bcsZ deletion mutant of S. Typhimurium MAE14 (UMR1 ΔcsgBA), the curli deficient S. Typhimurium derivative of UMR1. No alteration in the morphotype could be observed upon bcsZ deletion or overexpression after growth on Congo Red agar plates incubated at 37°C for 72 h. VC=pBAD30; pBcsZ=bcsZ cloned in pBAD30; pBcsZE56A=catalytic mutant of BcsZ cloned in pBAD30. [file 12934_2016_576_MOESM7_ESM.pdf]

Additional file 7

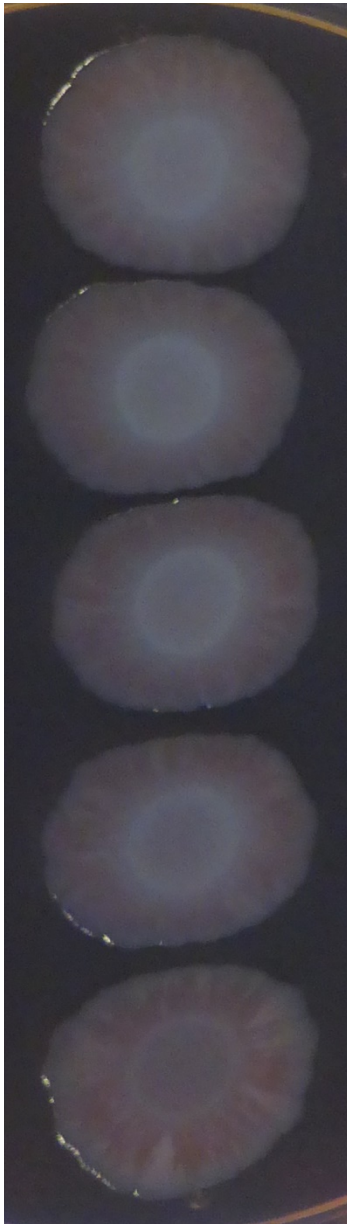

Δ*csgBA* **-VC**

Δ*csgBA* Δ*bcsZ* **-VC**

Δ*csgBA* Δ*bcsZ* **-pBcsZ**

Δ*csgBA* Δ*bcsZ* **–pBcsZ**$_{E56A}$

Δ*csgBA* Δ*bcsA-VC*
